# The biogeography of *Elaphe sauromates* (Pallas, 1814), with a description of a new rat snake species

**DOI:** 10.7717/peerj.6944

**Published:** 2019-05-28

**Authors:** Daniel Jablonski, Oleg V. Kukushkin, Aziz Avcı, Sabina Bunyatova, Yusuf Kumlutaş, Çetin Ilgaz, Ekaterina Polyakova, Konstantin Shiryaev, Boris Tuniyev, David Jandzik

**Affiliations:** 1 Department of Zoology, Comenius University in Bratislava, Bratislava, Slovakia; 2 Department of Biodiversity Studies and Ecological Monitoring, T. I. Vyazemski Karadag Research Station—Nature Reserve, Russian Academy of Sciences, Theodosia, Crimea; 3 Laboratory of Herpetology, Zoological Institute, Russian Academy of Sciences, Saint Petersburg, Russia; 4 Department of Biology, Faculty of Science and Arts, Adnan Menderes University, Aydın, Turkey; 5 Laboratory of Herpetology, Institute of Zoology, National Academy of Sciences of Azerbaijan, Baku, Azerbaijan; 6 Department of Biology, Faculty of Science, Dokuz Eylül University, Buca-İzmir, Turkey; 7 Research and Application Center for Fauna and Flora, Dokuz Eylül University, Buca-İzmir, Turkey; 8 Zoological Department, Tula State Regional Exotarium, Ministry of Culture of Tula Region, Tula, Russia; 9 Scientific Department, Federal State Institution Sochi National Park, Sochi, Russia; 10 Department of Zoology, Charles University in Prague, Prague, Czech Republic

**Keywords:** Anatolia, Diversification, Eurasia, Evolution, Phylogeny, Phylogeography, Reptiles, Taxonomy

## Abstract

**Background:**

The rat snake genus *Elaphe* once comprised several dozens of species distributed in temperate through tropical zones of the New and Old World. Based on molecular-genetic analyses in early 2000s, the genus was split into several separate genera, leaving only 15 Palearctic and Oriental species as its members. One of the three species also occurring in Europe is *Elaphe sauromates*, a robust snake from the Balkans, Anatolia, Caucasus, Ponto-Caspian steppes, and Levant that has been suspected to be composed of two or more genetically diverse populations. Here, we studied the genetic structure and morphological variation of *E. sauromates*, aiming to better understand its inter-population relationships and biogeography, and subsequently revise its taxonomy.

**Methods:**

We reconstructed the phylogeography and analyzed the genetic structure of *E. sauromates* populations originating from most of its geographic range using both mitochondrial (*COI*, *ND4*) and nuclear (*C-MOS*, *MC1R*, *PRLR*, *RAG1*) DNA gene fragments. We employed Maximum likelihood and Bayesian inference methods for the phylogenetic tree reconstructions, supplemented with species delimitation methods, analysis of haplotype networks, and calculation of uncorrected *p*-distances. Morphological variation in 15 metric and 18 meristic characters was studied using parametric univariate tests as well as multivariate general linearized models. In total, we analyzed sequences originating from 63 specimens and morphological data from 95 specimens of *E. sauromates* sensu lato.

**Results:**

The molecular phylogeny identified two clearly divergent sister lineages within *E. sauromates*, with both forming a lineage sister to *E. quatuorlineata*. The genetic distance between them (5.80–8.24% in mtDNA) is similar to the distances among several other species of the genus *Elaphe*. Both lineages are also moderately morphologically differentiated and, while none of the characters are exclusively diagnostic, their combination can be used for confident lineage identification. Here, following the criteria of genetic and evolutionary species concepts, we describe the lineage from eastern Anatolia and parts of the Lesser and Great Caucasus as a new species *E. urartica* sp. nov.

**Discussion:**

*Elaphe urartica* sp. nov. represents a cryptic species whose ancestors presumably diverged from their common ancestor with *E. sauromates* around the Miocene-Pliocene boundary. The intraspecific genetic structure indicates that the recent diversity of both species has been predominantly shaped by Pleistocene climatic oscillations, with glacial refugia mainly located in the Balkans, Crimea, and/or Anatolia in *E. sauromates* and Anatolia and/or the Caucasus in *E. urartica* sp. nov.

## Introduction

The Western Palearctic region is home to great diversity of herpetofauna (Sindaco & Jeremčenko, 2008; Sindaco, Venchi & Grieco, 2013). Among the most emblematic members are the rat snakes of the genus *Elaphe* Fitzinger in Wagler, 1833, once a large genus with distribution in temperate, subtropical, and tropical zones of both eastern and western hemisphere (Schulz, 1996). With the rise of molecular taxonomy in the last decades of the 20th century, the complicated relationships among dozens of species have been studied with a new rigor, resulting in the New-World as well as most Old-World species being identified as new or resurrected genera (Helfenberger, 2001; Lenk, Joger & Wink, 2001; Utiger et al., 2002; Chen et al., 2017). As a consequence, the genus *Elaphe* is comprised of 15 species (Wallach, Williams & Boundy, 2014; Chen et al., 2017), with most occurring exclusively in Asia. The high diversity in Asia supports the hypothesis of the origin of the genus in this region (Lenk, Joger & Wink, 2001; Utiger et al., 2002; Chen et al., 2017). Outside of Asia, three *Elaphe* species are also found in southern-eastern and eastern Europe: *Elaphe sauromates* (Pallas, 1814), *E. quatuorlineata* (Bonnaterre, 1790), the species with the western-most distribution of all *Elaphe*, and *E. dione* (Pallas, 1773) with mainly Asian distribution (Sindaco, Venchi & Grieco, 2013). *Elaphe sauromates* is also the type species of the genus *Elaphe*.

*Elaphe sauromates* is a robustly built snake whose biology has been systematically understudied and, despite its relatively large size, is only rarely encountered in the wild (Böhme & Ščerbak, 1993). It was described as a separate species under the name *Elaphis sauromates* (see: Strauch, 1873), followed by being synonymized with *Coluber quatuorlineatus* Bonnaterre, 1790, and subsequently treated as its subspecies *C. quatuorlineatus sauromates* (Boulenger, 1894). At the beginning of the 21st century, morphological and molecular-phylogenetic studies justified the species status of *E. sauromates* (Helfenberger, 2001; Lenk, Joger & Wink, 2001; Utiger et al., 2002) and this view has been largely accepted to date (Sindaco, Venchi & Grieco, 2013; Sillero et al., 2014; Wallach, Williams & Boundy, 2014; Jablonski, Soltys & Simonov, 2019). Aside from confirming the separate species status of *E. sauromates*, early genetic studies found a surprisingly high genetic distance between populations from Kazakhstan and eastern Turkey (5.0–6.5% in Lenk, Joger & Wink, 2001 or even 7.6% in Huang et al., 2012), which was quite similar to the distances between *E. sauromates* and *E. quatuorlineata*, *E. dione* and *E. bimaculata* Schmidt, 1925*, E. quadrivirgata* (Boie, 1826) and *E. carinata* (Günther, 1864), or between *E. quadrivirgata* and *E. schrenckii* (Strauch, 1873) (Utiger et al., 2002; Huang et al., 2012). In addition to genetic divergence, some morphological and color variation seems to be geographically correlated, indicating possible phenotypic differentiation of at least two genetic forms (Zinner, 1972; Tiedemann & Häupl, 1978; Schulz, 1996). Thus, there is a clear indication that the taxonomy of *E. sauromates* requires a revision based on a detailed study of both genetic and morphological variation.

Here, we studied the genetic structure and morphology of *E. sauromates* in biogeographic framework using robust geographic sampling, with the aim of elucidating the levels of divergence and phylogenetic relationships among the populations originating from most areas of the known taxon range. We analyzed sequences of mitochondrial and nuclear DNA fragments, snake measurements and pholidosis and have revealed the existence of cryptic diversity within *E. sauromates*. As a result, we describe a new species of a rat snake of the genus *Elaphe*.

## Material and Methods

### Tissue sampling, DNA extraction, and PCR amplification

**Figure 1:**
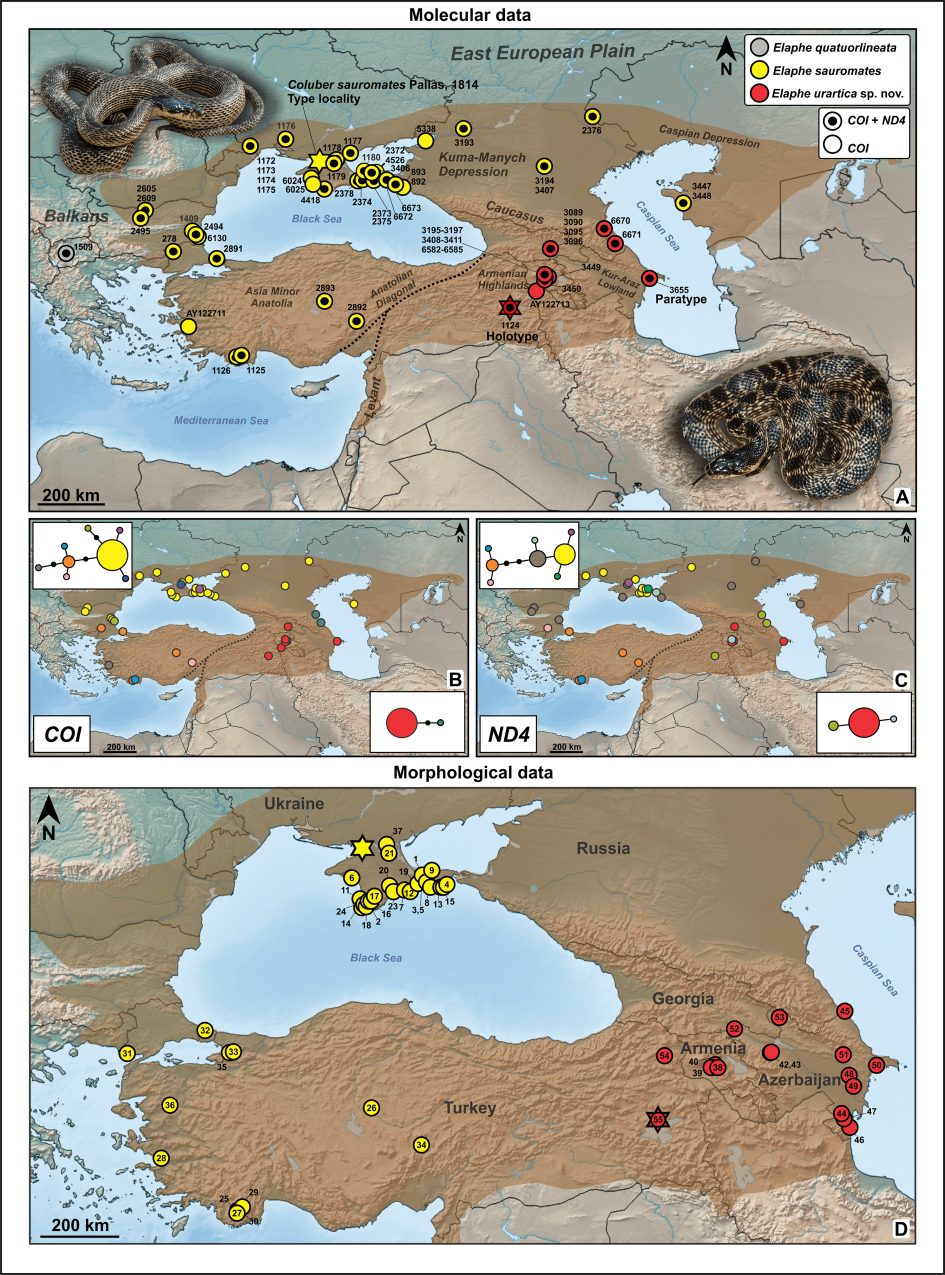
Geographic distribution of the samples used in the molecular-phylogenetic (A) and morphological (D) analyses. The colors correspond to the species and to individual *COI* and *ND4* haplotypes. Inset images (B and C) show the haplotype networks of both mtDNA genes analyzed. The stars indicate the type localities of *Elaphe sauromates* (yellow; photo by Mark Pestov) and *E. urartica* sp. nov. (red; photo by Ilya Korshunov). For more details on samples and localities, refer to Tables 1 and 2.

Between 2000 and 2017 we collected tissue samples of *E. sauromates* sensu lato (s.l.) from an area extending from the eastern Balkans to western Kazakhstan. We covered almost the entire distribution range except for the central-southern Turkey, northern Iran, Turkmenistan, Uzbekistan, and the Levant (Israel, Lebanon, Syria; Fig. 1). In total we obtained 90 tissue samples of *E. sauromates* s. l., and 63 of them were subsequently used in our molecular analyses. All sample information (DNA working codes, sampling localities, GenBank Accession Numbers, and comments) is presented in Table 1. We collected blood samples, ventral scales, and saliva from living specimens, while muscle and liver samples were collected from the dead (road-kills) or museum deposited individuals. All tissue samples were preserved in 96% ethanol or frozen and stored at −25 or −80 °C.

**Table 1:** List of the material used in the molecular-phylogenetic analyses.

Code	Taxon	Country	Locality	Coordinates		GenBank accession number						Museum voucher	References
				*N*	*E*	*COI*	*ND4*	*RAG1*	*PRLR*	*CMOS*	*MC1R*		
	*Oocatochus rufodorsatus*	–	–	–	–	NC_022146	NC_022146	–	–	–	–	–	Li et al. (2014)
	*Zamenis lineatus*	–	–	–	–	HQ392546	DQ902319	–	–	–	–	–	Musilová et al. (2011)/Burbrink & Lawson (2007)
LSUMZ 40626	*E. quatuorlineata*	–	–	–	–	–	–	–	–	AY486955	–	–	Nagy et al. (2004)
1509	*E. quatuorlineata*	Northern Macedonia	Crkvino	41.66	21.82	MK640236	MK640366	MK640344	MK640300	–	MK640325	–	This study
3195	*E. urartica*. sp. nov.	Armenia	Mt. Gutanasar, Abovyan	40.37	44.68	MK640260	MK640407	MK640362	MK640316	–	MK640341	TuRE ES 0000003301	This study
3196	*E. urartica*. sp. nov.	Armenia	Mt. Gutanasar, Abovyan	40.37	44.68	MK640261	MK640408	–	MK640317	–	–	TuRE ES 0000003300	This study
3197	*E. urartica*. sp. nov.	Armenia	Mt. Gutanasar, Abovyan	40.37	44.68	MK640262	MK640409	–	MK640318	–	–	–	This study
3408	*E. urartica*. sp. nov.	Armenia	Mt. Gutanasar, Abovyan	40.37	44.68	MK640263	MK640410	MK640363	–	–	MK640342	–	This study
3409	*E. urartica*. sp. nov.	Armenia	Mt. Gutanasar, Abovyan	40.37	44.68	MK640264	MK640411	–	–	–	–	–	This study
3410	*E. urartica*. sp. nov.	Armenia	Mt. Gutanasar, Abovyan	40.37	44.68	MK640265	MK640412	–	–	–	–	–	This study
3411	*E. urartica*. sp. nov.	Armenia	Mt. Gutanasar, Abovyan	40.37	44.68	MK640266	MK640413	–	–	–	–	–	This study
6582	*E. urartica*. sp. nov.	Armenia	Mt. Gutanasar, Abovyan	40.27	44.63	MK640270	MK640417	–	–	–	–	–	This study
6583	*E. urartica*. sp. nov.	Armenia	Mt. Gutanasar, Abovyan	40.27	44.63	MK640271	MK640418	–	–	–	–	–	This study
6584	*E. urartica*. sp. nov.	Armenia	Mt. Gutanasar, Abovyan	40.27	44.63	MK640272	MK640419	–	–	–	–	–	This study
6585	*E. urartica*. sp. nov.	Armenia	Mt. Gutanasar, Abovyan	40.27	44.63	MK640273	MK640420	–	–	–	–	–	This study
3449	*E. urartica*. sp. nov.	Armenia	Mt. Atis, Kotayikskoe Plateau	40.31	44.73	MK640267	MK640414	MK640364	–	–	–	TuRE ES 0000003303	This study
3450	*E. urartica*. sp. nov.	Armenia	Mt. Atis, Kotayikskoe Plateau	40.36	44.61	MK640268	MK640415	–	–	–	–	TuRE ES 0000003302	This study
3655	*E. urartica*. sp. nov. paratype	Azerbaijan	Guzdak, Qobustan	40.37	49.68	MK640269	MK640416	MK640365	–	–	MK640343	IZANAS T17	This study
1409	*E. sauromates*	Bulgaria	Pomorie	42.59	27.60	MK640290	–	–	–	–	–	–	This study
2494	*E. sauromates*	Bulgaria	Poda	42.44	27.46	MK640297	MK640385	–	MK640307	–	MK640335	–	This study
2495	*E. sauromates*	Bulgaria	Letnitsa	43.31	25.13	MK640298	MK640386	MK640350	–	–	–	–	This study
2605	*E. sauromates*	Bulgaria	Svistov	43.62	25.32	MK640237	MK640387	–	–	–	–	–	This study
2609	*E. sauromates*	Bulgaria	Svistov	43.62	25.31	MK640238	MK640388	MK640351	–	–	–	–	This study
6130	*E. sauromates*	Bulgaria	Djuni	42.36	27.71	MK640253	–	–	–	–	–	–	
1179	*E. sauromates*	“Crimea”	Solenoe Ozero	45.94	34.46	MK640288	MK640377	MK640348	MK640304	MK640322	MK640331	–	This study
1180	*E. sauromates*	“Crimea”	Cape Kazantip	45.46	35.84	MK640289	MK640378	–	–	–	–	–	This study
2372	*E. sauromates*	“Crimea”	Zolotoe, Kerch	45.40	36.12	MK640291	MK640379	–	–	–	–	–	This study
2373	*E. sauromates*	“Crimea”	Yuzhnoe	45.13	35.60	MK640292	MK640380	–	–	–	–	–	This study
2374	*E. sauromates*	“Crimea”	Primorskyi	45.13	35.59	MK640293	MK640381	–	–	–	–	–	This study
2375	*E. sauromates*	“Crimea”	Opuk	45.08	36.29	MK640294	MK640382	–	MK640305	MK640323	MK640332	–	This study
2378	*E. sauromates*	“Crimea”	Yarkoe	45.14	35.74	MK640296	MK640384	MK640349	–	–	MK640334	–	This study
3406	*E. sauromates*	“Crimea”	Cape Kazantip II	45.47	35.85	MK640244	MK640394	MK640356	–	–	MK640338	–	This study
4418	*E. sauromates*	“Crimea”	Nauchnyi	44.73	34.01	MK640248	MK640398	–	–	–	–	–	This study
4526	*E. sauromates*	“Crimea”	Yurkino	45.44	36.57	MK640249	MK640399	–	–	–	–	–	This study
6024	*E. sauromates*	“Crimea”	Kara–Tobe	45.13	33.62	MK640251	–	–	–	–	–	–	This study
6025	*E. sauromates*	“Crimea”	Nikolaevka	44.97	33.60	MK640252	–	–	–	–	–	–	This study
3089	*E. urartica*. sp. nov.	Georgia	Tbilisi	41.69	44.80	MK640256	MK640403	MK640359	MK640312	–	MK640340	–	This study
3090	*E. urartica*. sp. nov.	Georgia	Tbilisi	41.69	44.80	MK640257	MK640404	–	MK640313	–	–	–	This study
3095	*E. urartica*. sp. nov.	Georgia	Tbilisi	41.69	44.80	MK640258	MK640405	MK640360	MK640314	–	–	–	This study
3096	*E. urartica*. sp. nov.	Georgia	Tbilisi	41.69	44.80	MK640259	MK640406	MK640361	MK640315	–	–	–	This study
3447	*E. sauromates*	Kazakhstan	Mangystau Peninsula	43.60	51.57	MK640246	MK640396	MK640358	–	–	MK640339	–	This study
3448	*E. sauromates*	Kazakhstan	Mangystau Peninsula	43.60	51.57	MK640247	MK640397	–	–	–	–	–	This study
892	*E. sauromates*	Russia	Novorossijsk	44.70	37.73	MK640277	–	–	–	–	–	–	This study
893	*E. sauromates*	Russia	Novorossijsk	44.70	37.73	MK640278	–	–	–	–	–	–	This study
2376	*E. sauromates*	Russia	Bol’shoy Bogdo Mt.	48.14	46.86	MK640295	MK640383	–	MK640306	–	MK640333	–	This study
3193	*E. sauromates*	Russia	Razdorskaya	47.54	40.64	MK640242	MK640392	MK640354	–	–	MK640337	–	This study
3194	*E. sauromates*	Russia	Iki–Burul, Kalmykia	45.72	44.56	MK640243	MK640393	MK640355	MK640310	–	–	–	This study
3407	*E. sauromates*	Russia	Iki–Burul, Kalmykia	45.72	44.56	MK640245	MK640395	MK640357	–	–	–	–	This study
5338	*E. sauromates*	Russia	Taganrogskyi Gulf	46.99	39.01	MK640250	–	–	–	–	–	ZISP 26197	This study
6670	*E. urartica*. sp. nov.	Russia (Dagestan)	Leninkent	42.96	47.35	MK640274	MK640421	–	–	–	–	ZM MSU 10784	This study
6671	*E. urartica*. sp. nov.	Russia (Dagestan)	Yersi	42.02	48.01	MK640275	MK640422	–	–	–	–	ZM MSU 11043	This study
6672	*E. sauromates*	Russia	Varvarovka	44.82	37.38	MK640254	MK640400	–	–	–	–	ZM MSU 14729	This study
6673	*E. sauromates*	Russia	Varvarovka	44.77	37.39	MK640255	MK640401	–	–	–	–	ZM MSU 14731	This study
278	*E. sauromates*	Turkey	Iskenderun	41.63	26.68	MK640276	MK640367	MK640345	MK640301	MK640319	MK640326		This study
1124	*E. urartica*. sp. nov. holotype	Turkey	Kısıklı, Süphan Mts., Bitlis	38.93	42.91	MK640299	MK640402	–	MK640311	MK640324	–	ZDEU 26/2012	This study
1125	*E. sauromates*	Turkey	Akcay–Elmalı	36.55	29.79	MK640279	MK640368	–	MK640302	MK640320	MK640327	ZDEU 108/2011	This study
1126	*E. sauromates*	Turkey	Göltara, Elmalı	36.56	29.89	MK640280	MK640369	–	MK640303	MK640321	MK640328	ZDEU 298/2013	This study
2891	*E. sauromates*	Turkey	İhsaniye–Eyüp, İstanbul	41.28	28.75	MK640239	MK640389	–	–	–	–	ZDEU 180/2014	This study
2892	*E. sauromates*	Turkey	Öksüt Village–Develi	38.28	35.51	MK640240	MK640390	MK640352	MK640308	–	MK640336	ZDEU 15/2015	This study
2893	*E. sauromates*	Turkey	Yağmurlusayobası	39.25	33.94	MK640241	MK640391	MK640353	MK640309	–	–	ZDEU 14/2015	This study
SH556	*E. urartica*. sp. nov.	Turkey	Mt. Ararat	39.68	44.20	AY122713	–	–	–	–	–	–	Utiger et al. (2002)
SH972	*E. sauromates*	Turkey	Selçuk	37.95	27.37	AY122711	–	–	–	–	–	–	Utiger et al. (2002)
1172	*E. sauromates*	Ukraine	Egorivka	46.71	30.40	MK640281	MK640370	–	–	–	–	–	This study
1173	*E. sauromates*	Ukraine	Egorivka	46.71	30.40	MK640282	MK640371	–	–	–	–	–	This study
1174	*E. sauromates*	Ukraine	Egorivka	46.71	30.40	MK640283	MK640372	–	–	–	–	–	This study
1175	*E. sauromates*	Ukraine	Egorivka	46.71	30.40	MK640284	MK640373	–	–	–	–	–	This study
1176	*E. sauromates*	Ukraine	Zayichevske	47.04	32.09	MK640285	MK640374	MK640346	–	–	MK640329	–	This study
1177	*E. sauromates*	Ukraine	Davydovka	46.37	35.18	MK640286	MK640375	MK640347	–	–	MK640330	–	This study
1178	*E. sauromates*	Ukraine	Chongar	46.02	34.50	MK640287	MK640376	–	–	–	–	–	This study

**Note:**

Museum abbreviations: IZANAS, Institute of Zoology, Azerbaijan, National Academy of Sciences, Baku, Republic of Azerbaijan; TuRE, Zoological Department of Tula State Regional Exotarium, Ministry of Culture of Tula Region, Tula, Russia; ZDEU, Zoology Department of Ege University, Turkey; ZISP, Institute of Zoology, Russian Academy of Sciences, Saint Petersburg, Russia; ZMMSU, Zoological Museum of Moscow State University, Moscow, Russia.

Total genomic DNA was extracted using the NucleoSpin Tissue kit (MACHEREY—NAGEL, Duren, Germany), following the manufacturer’s instructions. Sequences of two mitochondrial (mtDNA) and four nuclear DNA (nDNA) genes were targeted: the DNA barcoding segment of cytochrome c oxidase subunit 1 (*COI*), the mitochondrial protein-coding segment of NADH dehydrogenase subunit 4 (*ND4*) (including the flanking tRNAs Serine, Histidine, and part of Leucine), and the nuclear protein-coding genes for the prolactin receptor (*PRLR*), the oocyte maturation factor Mos (*C-MOS*), recombination activation gene 1 (*RAG1*), and melanocortin 1 receptor (*MC1R*). Primers used for PCR amplifications are listed in Table S1. All amplification procedures involved an initial cycle of denaturation at 94 °C for 3–7 min, 35–40 subsequent cycles at 94, 48–63, and 72 °C for 1 min each, followed by a final extension step at 72 °C for 5–10 min. PCR products were purified with ExoSAP-IT™ PCR Product Cleanup Reagent (USB Europe GmbH, Staufen, Germany; manufacturer’s protocol). Sequencing was performed by Macrogen Inc. (Seoul, South Korea or Amsterdam, Netherlands; http://www.macrogen.com) with the ABI PRISM 3100 capillary sequencer using the PCR amplification primers. All newly obtained sequences have been deposited in GenBank (Table 1).

### Alignment, genetic divergence and model selection

DNA sequences were manually checked, aligned, and inspected using BioEdit 7.0.9.0 (Hall, 1999). No stop codons were detected with into amino acid translated sequences using the vertebrate genetic code in the program DnaSP 5.10 (Librado & Rozas, 2009). The same program was used to calculate uncorrected *p*-distances among the main clades and to estimate the haplotype diversity (*Hd*), number of segregating sites (*S*), variables (*V*), nucleotide diversity (*π*), and parsimony informative (*Pi*) sites for the selected groups. Heterozygous positions in nuclear genes were manually identified based on the presence of double peaks in chromatograms. Identified heterozygous loci were coded according to the IUPAC ambiguity codes. The best-fit codon-partitioning schemes and the best-fit substitution models for phylogenetic analyses with concatenated dataset (*COI* 581 bp + *ND4* 810 bp) were selected using PartitionFinder v1.1.1. (Lanfear et al., 2012) with the following parameters: Bayesian approach (BA)—linked branch length; all models; BIC model selection; greedy schemes search; data blocks by codons for each used marker. The best partitioning scheme and models of nucleotide substitutions were: first and second positions (HKY+G), third position (K80). The Maximum likelihood (ML) analysis followed the same approach; the best substitution model in this case was GTR+G+I with a single partition.

### Phylogenetic and haplotype network analysis

Concatenated (*COI* + *ND4*) mitochondrial phylogenetic trees were inferred using the BA performed with MrBayes 3.2.1 (Ronquist et al., 2012) and ML analysis performed with RAxML 8.0 (Stamatakis, 2014). The BA analysis was set as follows; two separate runs, with four chains for each run, 10 million generations with trees sampled every 100th generation. The first 20% of trees were discarded as the burn–in after inspection for stationarity of log–likelihood scores of sampled trees in Tracer 1.6 (Rambaut et al., 2013; all parameters had effective sample sizes (ESSs) of >200). A majority-rule consensus tree was drawn from the post-burn-in samples and posterior probabilities were calculated as the frequency of samples recovering any particular clade. Nodes with posterior probability values ≥ 0.95 were considered as strongly supported. The ML clade support was assessed by 1,000 bootstrap pseudoreplicates. Sequences of other, closely related Eurasian rat snakes, *Oocatochus rufodorsatus* (Cantor, 1842) and *Zamenis lineatus* (Camerano, 1891) (for GenBank accession numbers see Table 1), were used in the analyses included as outgroups (Utiger et al., 2002; Chen et al., 2017).

In order to obtain better support for putative species boundaries of the divergent lineages of *Elaphe*, we employed three species delimitation methods: (i) Bayesian implementation of the Poisson tree processes model (bPTP; Zhang et al., 2013, https://species.h-its.org/), (ii) multi-rate Poisson Tree Processes (mPTP; Kapli et al., 2016) model, using the webserver (http://mptp.h-its.org/), and (iii) the general mixed yule-coalescent model (GMYC; Pons et al., 2006). For species delimitations analysis we used as input an ultrametric tree of mtDNA haplotypes constructed with BEAST 2.1 (Bouckaert et al., 2014). BEAST analyses were run under the uncorrelated log-normal relaxed clock approach with a Yule tree prior. Two independent runs were conducted with a chain length of 5 × 10^7^ iterations. Tracer 1.6 (Rambaut et al., 2013) was used to check for convergence of the chains and adequate ESSs. Independent runs were combined using LogCombiner (part of the BEAST package), discarding the first 25% of each run as burn-in. The maximum clade credibility tree was summarized with TreeAnnotator (part of the BEAST package) and visualized with Fig-Tree 1.4 (http://tree.bio.ed.ac.uk/software/figtree). GMYC species delimitation was conducted using the “splits” package (Ezard, Fujisawa & Barraclough, 2009) in R (R Development Core Team, 2019) under the single-threshold method.

The genealogical relationships between haplotypes of mtDNA and each nDNA marker were separately assessed with haplotype networks. For the purpose of allele network construction, sequences with more than one heterozygous site were resolved in PHASE 2.1.1 (Stephens, Smith & Donnelly, 2001) for which the input data were prepared in SeqPHASE (Flot, 2010). PHASE was run under default settings except for the probability threshold, which was set to 0.7. Haplotype networks of all analyzed markers were examined and drawn using PopArt (http://popart.otago.ac.nz) and the implemented parsimony network algorithm of TCS (Clement, Posada & Crandall, 2000), with 95% connection limit. Independent networks are considered distinct evolutionarily significant units, following Fraser & Bernatchez (2001), thus this analysis was also used to infer genetic structure within the studied taxa.

### Material for morphological analyses and collection of specimens

In total, we examined external morphology of 95 specimens (46 males, 29 females, 20 individuals of unidentified sex) of *E. sauromates* s. l. from the Crimean Peninsula, Turkey, Armenia, and Azerbaijan (Fig. 1D; Table 2). Only snakes with SVL ≥ 650 mm were used in the analyses of metric characters (75 adult animals in total), while all available specimens with known sex were used in scale count descriptive statistics and comparisons. The material was obtained either directly in the field or from collections of the following institutions: Zoology Department of Ege University, Turkey (ZDEU); Institute of Zoology, Azerbaijan, National Academy of Sciences, Baku, Republic of Azerbaijan (IZANAS); Institute of Zoology, Russian Academy of Sciences, Saint Petersburg, Russia (ZISP); Zoological Department of Tula State Regional Exotarium, Ministry of Culture of Tula Region, Tula, Russia (TuRE).

**Table 2:** List of the material used for morphological analyses.

Species	Country	Locality	Locality number	*N*	*E*	Elevation (m)	No. of adult specimens (males/females/unidentified)	No. of juvenile specimens (males/females/unidentified)	Museum voucher number
*E. sauromates*	“Crimea”	Akmonayi Isthmus, Kerch Peninsula	1	45.31	35.60	17	1/0/0	0/0/0	
*E. sauromates*	“Crimea”	Albat, Bakhchisarayi	2	44.64	33.91	343	2/2/2	0/0/0	
*E. sauromates*	“Crimea”	Between Bash-Kirghi and Dzhaga-Setdzhevyut, Kerch Peninsula	3	45.14	35.72	60	0/0/0	0/0/1	
*E. sauromates*	“Crimea”	Between Cape Opuk and Cape Kyz-Aul, Kerch Peninsula	4	45.06	36.30	7	1/0/0	0/0/0	
*E. sauromates*	“Crimea”	Between Hafuz and Dzhaga-Setdzhevyut, Kerch Peninsula	5	45.14	35.61	21	1/0/0	0/0/0	
*E. sauromates*	“Crimea”	Between Kara-Tobe and Nikolaevka	6	45.13	33.62	17	0/0/0	1/1/0	
*E. sauromates*	“Crimea”	Between Suuk-Su and Kiziltash, Sudak	7	44.93	35.00	167	0/0/0	0/1/0	
*E. sauromates*	“Crimea”	Cape Chauda, Kerch Peninsula	8	45.01	35.84	21	1/2/1	0/0/4	
*E. sauromates*	“Crimea”	Cape Kazantip, Kerch Peninsula	9	45.46	35.86	74	2/2/0	0/0/0	
*E. sauromates*	“Crimea”	captive breeding: female from Azov Sea coast, male from Black Sea coast, Kerch Peninsula	10	?	?	?	0/0/0	1/0/0	
*E. sauromates*	“Crimea”	Inkerman, upland Mekenzievy Gory, Sevastopol	11	44.66	33.59	75	0/1/0	0/0/0	
*E. sauromates*	“Crimea”	Karadag Reserve, Theodosia	12	44.91	35.20	3	1/0/0	0/0/1	
*E. sauromates*	“Crimea”	Lake Koyashskoe, Kerch Peninsula	13	45.06	36.18	3	0/0/0	0/0/1	
*E. sauromates*	“Crimea”	Mt. Kalafatlar near Cape Aya, Sevastopol	14	44.47	33.67	401	1/0/0	0/0/0	
*E. sauromates*	“Crimea”	Mt. Opuk, Kerch Peninsula	15	45.03	36.22	54	2/1/0	0/0/2	
*E. sauromates*	“Crimea”	Nauchnyi	16	44.73	34.01	549	0/0/0	1/0/0	
*E. sauromates*	“Crimea”	Orta-Sabla, reservoir Partizanskoe, Simferopol	17	44.82	34.06	304	0/1/0	0/0/0	
*E. sauromates*	“Crimea”	Peredovoe (Urkusta), Bayidar Valley, Sevastopol	18	44.52	33.81	370	0/1/0	0/0/0	
*E. sauromates*	“Crimea”	Primorskyi, Akmonayiskyi Isthmus, Theodosia	19	45.13	35.45	33	0/0/1	0/0/0	
*E. sauromates*	“Crimea”	Sary-Su near Belogorsk (Karasu-Bazar)	20	45.04	34.54	330	0/1/0	0/0/0	
*E. sauromates*	“Crimea”	Solenoe Ozero railway station, Dzhankoyi	21	45.92	34.49	11	1/1/3	0/0/0	
*E. sauromates*	“Crimea”	Tatar-Koyi, Mt. Kyz-Kermen, Bakhchisarayi	22	44.70	33.92	210	1/0/0	0/0/0	
*E. sauromates*	“Crimea”	Tiup-Tarkhan Peninsula, Dzhankoyi	23	44.87	34.67	3	0/0/2	0/0/0	
*E. sauromates*	“Crimea”	Uppa-Koyi, ruines of aul Uzen-Bash, Sevastopol	24	44.55	33.77	397	1/0/0	0/0/0	
*E. sauromates*	Turkey	Akçay-Elmalı	25	36.55	29.79	1,477	0/0/0	1/0/0	
*E. sauromates*	Turkey	Yağmurlusayobası	26	39.25	33.94	1,412	1/0/0	0/0/0	
*E. sauromates*	Turkey	Çığlıkara-Elmalı	27	36.55	29.88	1,690	1/0/0	0/0/0	
*E. sauromates*	Turkey	Efes-Selçuk	28	37.94	27.37	15	1/0/0	0/0/0	
*E. sauromates*	Turkey	Elmalı	29	36.73	29.90	1,086	0/1/0	0/0/0	
*E. sauromates*	Turkey	Göltarla, Elmalı	30	36.56	29.89	1,690	1/0/0	0/0/0	
*E. sauromates*	Turkey	Hasköy-Enez	31	40.66	26.34	62	1/0/0	0/0/0	
*E. sauromates*	Turkey	İhsaniye-Eyüp, İstanbul	32	41.28	28.75	20	1/0/0	0/0/0	
*E. sauromates*	Turkey	Karamürsel	33	40.68	29.60	21	1/0/0	0/0/0	
*E. sauromates*	Turkey	Öksüt Village-Develi	34	38.28	35.51	1,685	1/0/0	0/0/0	
*E. sauromates*	Turkey	Tokmak Village-Karmürsel	35	40.68	29.54	42	0/1/0	0/0/0	
*E. sauromates*	Turkey	Yağcılı Village-Soma	36	39.33	27.66	348	1/0/0	0/0/0	
*E. sauromates*	Ukraine	Island Kuyuk-Tuk	37	46.12	34.42	7	0/0/0	0/0/1	
***E. urartica* sp. nov., paratype**	Armenia	Mt. Atis, Kotayikskoe Plateau	38	40.31	44.73	2,345	0/1/0	0/0/0	TuRE ES 0000003303
***E. urartica* sp. nov., paratype**	Armenia	Mt. Atis, Kotayikskoe Plateau	39	40.36	44.61	1,456	1/0/0	0/0/0	TuRE ES 0000003302
***E. urartica* sp. nov., paratypes**	Armenia	Mt. Gutanasar, Gegamsky Ridge, near Abovyan	40	40.37	44.69	2,300	1/1/0	3/5/0	TuRE ES 0000003300; TuRE ES 0000003301
*E. urartica* sp. nov.	Azerbaijan	Azerbaijan—locality unknown	41	?	?	?	2/0/0	0/0/0	
*E. urartica* sp. nov.	Azerbaijan	Ganja	42	40.68	46.36	430	1/0/0	1/1/0	
*E. urartica* sp. nov.	Azerbaijan	Ganja, “near Areshsk”	43	40.69	46.37	450	1/0/0	0/0/0	
*E. urartica* sp. nov.	Azerbaijan	Goytapa	44	39.12	48.60	0	1/0/0	0/0/0	
*E. urartica* sp. nov.	Azerbaijan	Khazar	45	41.76	48.70	0	0/1/0	0/0/0	
***E. urartica* sp. nov., paratype**	Azerbaijan	Lenkoran	46	38.75	48.85	0	1/0/0	0/0/0	IZANAS 68
*E. urartica* sp. nov.	Azerbaijan	Masally	47	39.02	48.67	0	1/0/0	0/0/0	
*E. urartica* sp. nov.	Azerbaijan	Mughan	48	40.10	48.84	0	1/0/0	0/0/0	
*E. urartica* sp. nov.	Azerbaijan	Qaraçala	49	39.82	48.95	0	0/1/0	0/0/0	
***E. urartica* sp. nov., paratype**	Azerbaijan	Guzdak, Qobustan	50	40.37	49.68	140	1/0/0	0/0/0	IZANAS T-17
***E. urartica* sp. nov., paratypes**	Azerbaijan	Şamaxi	51	40.63	48.63	648	1/1/0	0/0/0	IZANAS 69 IZANAS 70
***E. urartica* sp. nov., paratype**	Azerbaijan	Zivi-Zkaro (Tsivi-Tskaro)	52	41.32	45.27	246	0/1/0	0/0/0	IZANAS 71
***E. urartica* sp. nov., paratypes**	Azerbaijan	Zaqatala	53	41.63	46.65	511	1/1/0	0/0/0	IZANAS 518 IZANAS 529
***E. urartica* sp. nov., paratype**	Turkey	Hoşerenler Plateau, Kars	54	40.61	43.09	1,747	1/0/0	0/0/0	ZDEU 114/1970
***E. urartica* sp. nov., holotype**	Turkey	Kısıklı, Süphan Mts.	55	38.93	42.91	2,394	1/0/0	0/0/0	ZDEU 26/2012

**Note:**

Specimens of the type series are in bold.

Field permits for this study were issued by the Republic of Turkey Ministry of Forest and Water Affairs (No. B.23.0.DMP.0.15.01–510–38417) and by the Crimean authority under the title “Study of Biodiversity and Landscape Structure of the Southeastern Crimea, monitoring of Biotic and Abiotic Components of the Regional Ecosystems” (FASO: AAA–A16–116022510087–5) and by “Study of the structure and dynamics of land ecosystems in various climatic zones” No. AAAA–A19–119012490044–3). All efforts were made to minimize animal suffering.

### Analysis of the external morphology

Body and tail lengths were measured with a ruler to the nearest one mm, while the head and individual skin plates were measured with a caliper with accuracy to the nearest 0.01 mm. For the purpose of obtaining basic information, the head scales of the juvenile snakes from Armenia were measured using their photographs (see Kropachev et al., 2015), but they were not used in further statistical analyses.

In total, we targeted 15 metric and 18 meristic (scale count) characters, however not all measurements or counts were available for each individual (see Sahlean et al., 2016; Table S2 for explanation of the characters). In addition to the measurements and scale counts, we calculated two main morphometric indices: the snout-vent length to tail length (SVL/TL) and the snout-vent length to head length (SVL/HL) ratio.

Prior to morphological analyses, the snakes were split into two groups based on the results of molecular-genetic analyses and their origin: *E. sauromates* sensu stricto (s. s.; 63 specimens) and a new species that is formally described and named below (32 specimens). All measures were a priori log-transformed and the homoscedasticity (equal variances) was checked by Levene’s test before the comparisons were carried out. We used independent sample *t*-tests to compare the length (SVL and total) of both snake groups. The tail and head lengths were compared using multivariate analysis of variance with SVL as a co-variate (MANCOVA). The overall head size was further compared with MANCOVA with head length used as a co-variate. The scale counts, which are not dependent on the snake length, were compared with multivariate analysis of variance (MANOVA). In all tests, the sexes were treated separately. We also examined the sexual differences within both groups and employed the same statistical strategy as in the species comparisons. The significance level was set to 0.05 and Bonferroni correction was applied in multiple-comparison analyses. All analyses were performed in SPSS Statistics 17.0.0. (SPSS Inc., Chicago, IL, USA).

### The type material note

While the holotype of the newly described species (see below) as well as eight paratypes are deposited in public museums or scientific institutions (see Tables 1 and 2), four paratypes are currently still alive at TuRE (as of March 2019; Tula, Russia). These individuals have been described in detail, photographed, and assigned identification numbers. Upon death, the animals will be fixed and deposited in one of the central zoological museums in Russia (ZISP and/or ZM MSU). While the use of live animals as type material is not recommended by some authors (Dubois & Nemésio, 2007), such practice is not prohibited by International Commission on Zoological Nomenclature (ICZN). We decided to include these specimens (originating from Armenia) among the type series to provide a better representation of the material that was predominantly used as tissue sample source for molecular-genetic analyses.

### Nomenclatural note

The electronic version of this article in portable document format will represent a published work according to the ICZN, and hence the new names contained in the electronic version are effectively published under that Code from the electronic edition alone. This published work and the nomenclatural acts it contains have been registered in ZooBank, the online registration system for the ICZN. The ZooBank Life Science Identifiers (LSIDs) can be resolved and the associated information viewed through any standard web browser by appending the LSID to the prefix http://zoobank.org/. The LSID for this publication is: urn:lsid:zoobank.org:pub:F0BF1D63–7BD5–4340–85A0–E044CC56CD5B. The online version of this work is archived and available from the following digital repositories: PeerJ, PubMed Central and CLOCKSS.

## Results

### Phylogeography and genetic diversity

**Figure 2:**
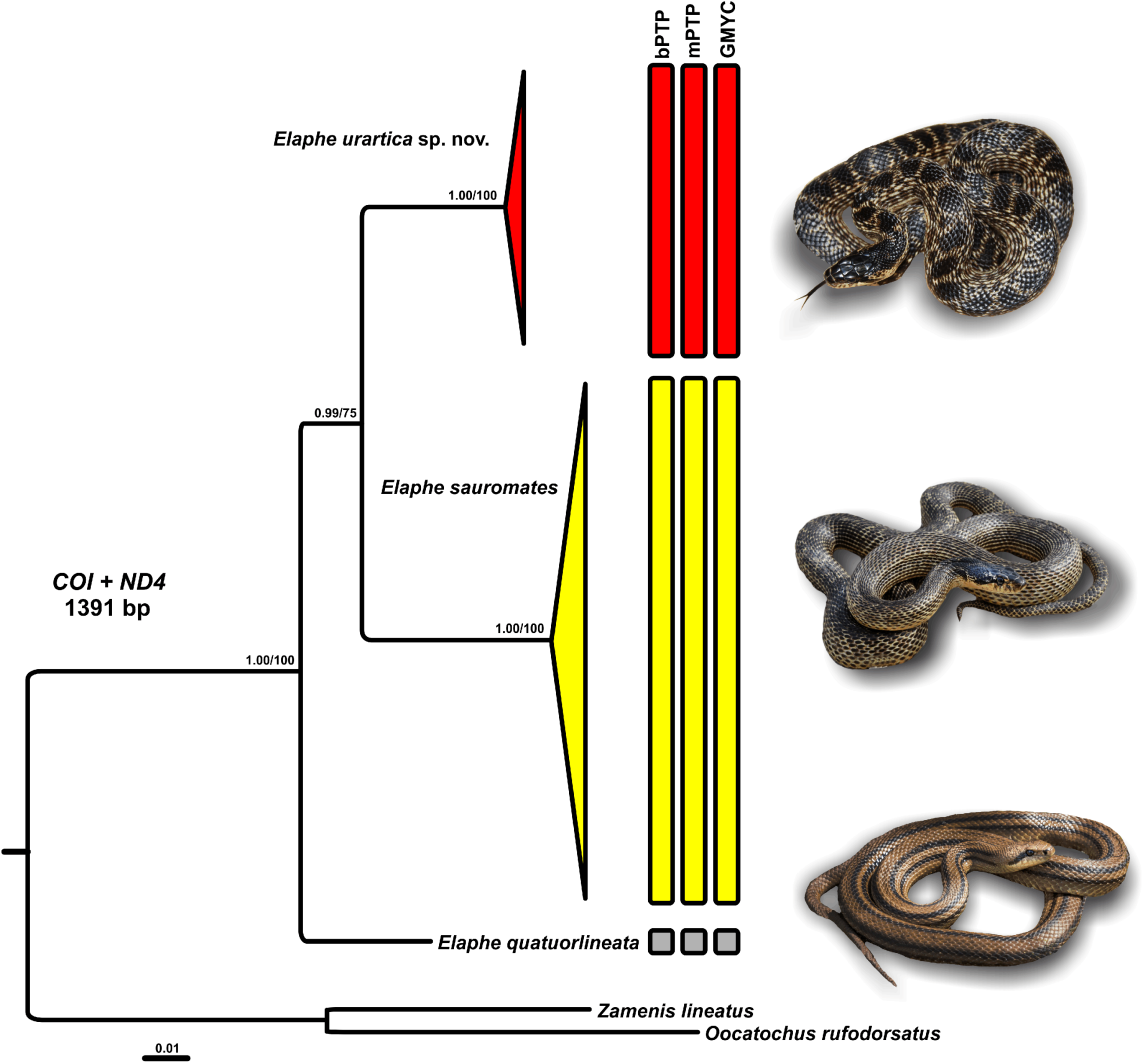
Phylogenetic relationships of *Elaphe quatuorlineata* (photo by Daniel Jablonski), *E. sauromates* (photo by Mark Pestov), and *E. urartica* sp. nov. (photo by Ilya Korshunov) reconstructed using Bayesian inference of concatenated *COI* and *ND4* sequences. The numbers above the branches represent Bayesian Posterior Probabilities/Bootstraps showing branch supports. The vertical bars next to the clades illustrate support of three mtDNA species delimitation methods for the three-species arrangement: the Bayesian implementation of the Poisson tree processes model (bPTP), the multi-rate Poisson Tree Processes (mPTP), and the Bayesian implementation of the general mixed yule-coalescent model (GMYC). The full BI and ML and trees that formed the basis for the presented tree can be found in Figs. S1 and S2.

The final nucleotide dataset of 4,068 bp comprised sequences of two mtDNA genes: *COI* (581 bp), *ND4* (810 bp), and fragments of four nuclear genes: *C-MOS* (459 bp), *MC1R* (650 bp), *PRLR* (552 bp), and *RAG1* (1,016 bp). The phylogenetic trees (ML and BA) of mtDNA concatenated dataset showed the same topologies with very similar, high statistical supports for each node (Fig. 2; Figs. S1 and S2). Therefore, we only present the BA tree here to show the topology and interrelationships among the studied snake lineages.

We identified two major, deeply divergent clades within *E. sauromates* s. l. Together they form a sister clade to that of *E. quatuorlineata* sequences. Uncorrected *p*-distances (mtDNA) among the clades ranged between 5.80% (*ND4*) and 8.24% (*COI*; Table 3). One of these clades corresponds to *E. sauromates* s. s., while the other one represents a distinct phylogenetic lineage, which we consider a new, separate species. The clade corresponding to *E. sauromates* has been detected (based on mtDNA) from western and central Anatolia (Turkey), the Balkans (Bulgaria, Turkey), southern Ukraine, Crimean Peninsula, southern Russia, and western Kazakhstan. Samples from the new species originated from eastern Anatolia (Turkey), Armenia, Azerbaijan, Georgia, and Dagestan (southern Russia). Both species seem to be geographically separated, presumably by the Anatolian Diagonal, and partially, also by the Great Caucasus (Fig. 1).

**Table 3:** Uncorrected *p*-distances (in %) in mtDNA (*COI/ND4*) and nDNA (CMOS/MC1R/PRLR/RAG1) sequences of *E. quatuorlineata*, *E. sauromates*, and *E. urartica* sp. nov.

	*E. quatuorlineata*	*E. sauromates*	*E. urartica* sp. nov.
*E. quatuorlineata*	–	0/0/0.73/0.30	0.15/0.15/0/0.20
*E. sauromates*	7.71/7.90	*0.57/0.42*	0.15/0.15/0.73/0.24
*E. urartica* sp. nov.	8.24/5.80	7.20/6.91	*0.52/0.16*

**Note:**

Intraspecific mtDNA (*COI*/*ND4*) average *p*-distance is in diagonal and italics.

All three methods of species delimitation, that is, bPTP, mPTP, and GMYC recognized the three divergent lineages as distinct entities among the analyzed members of *Elaphe* (Fig. 1): *E. quatuorlineata*, *E. sauromates*, and the new species described below that has a sister relationship to *E. sauromates* (Fig. 2).

The mtDNA diversity is lower in the new species than in *E. sauromates* s. s. We identified eight haplotypes in both *COI* and *ND4* of *E. sauromates* s. s., with the following characteristics of DNA polymorphism: *Hd* = 0.409; π = 0.179%; *S* = 10; *V* = 10; *Pi* = 2 (*COI*), *Hd* = 0.726; π = 0.207%; *S* = 9; *V* = 9; *Pi* = 4 (*ND4*). In the new species, we only detected two (*COI*) and three haplotypes (*ND4*), respectively, with lower values describing DNA polymorphism: *Hd* = 0.173; π = 0.067%; *S* = 2; *V* = 2; *Pi* = 2 (*COI*), *Hd* = 0.279; π = 0.036%; *S* = 2; *V* = 2; *Pi* = 1 (*ND4*). Neither clade shows a clear geographic structure based on the haplotype networks (Figs. 1B and 1C), although *E. sauromates* s. s. is more diversified. Haplotypes of both *E. sauromates* s. s. mtDNA genes are more broadly distributed, mainly in the northern Black Sea region. Unique haplotypes of this clade were detected in central and southwestern Anatolia, Bulgaria, and Crimea. In contrary, the new species shows much lower haplotype diversity with unique haplotypes found in Dagestan (*COI*, *ND4*), Armenia, and eastern Anatolia (*ND4*) (Fig. 1).

**Figure 3:**
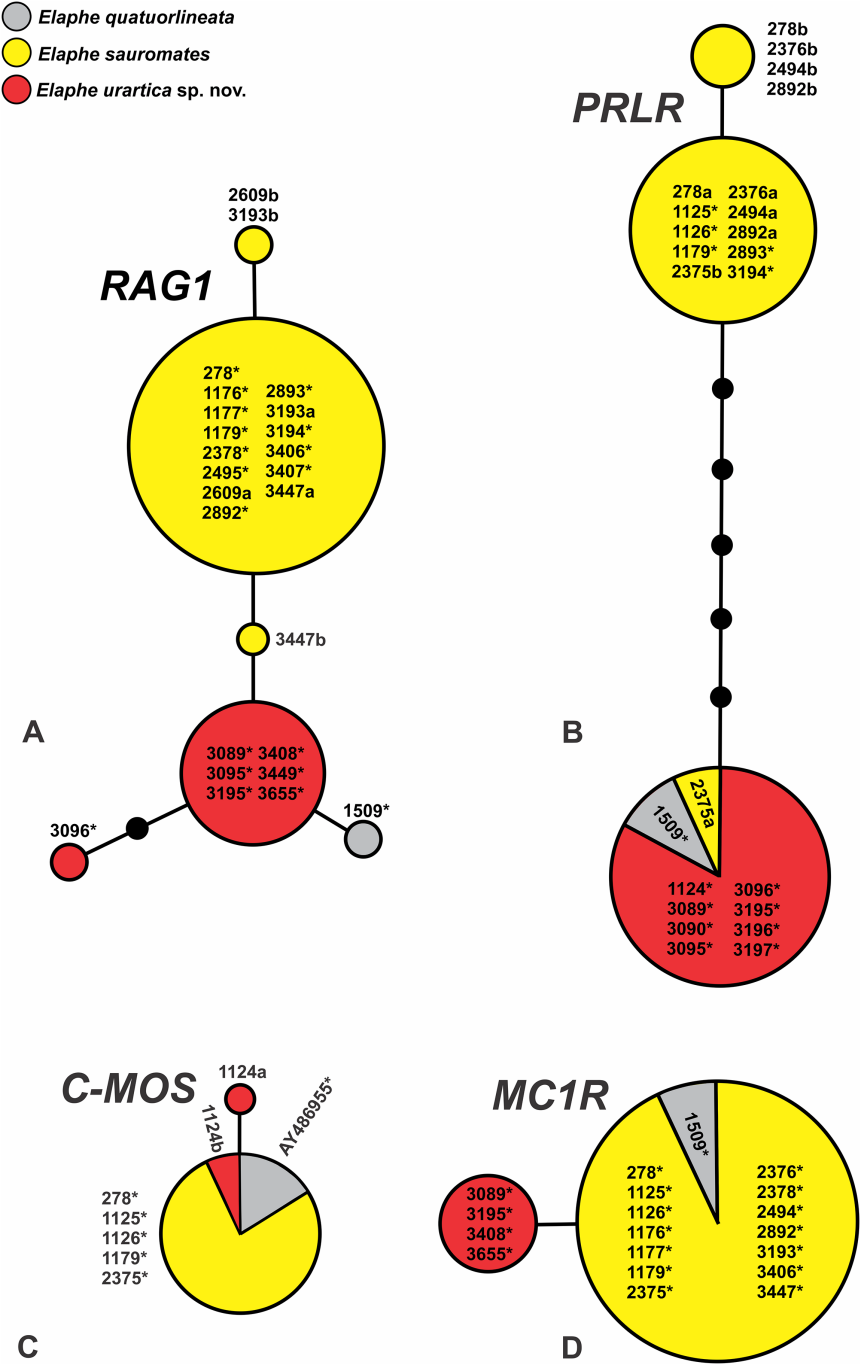
Nuclear allele network of the phased sequences of *RAG1* (A), *PRLR* (B), *C-MOS* (C), and *MC1R* (D) of *Elaphe quatuorlineata* (gray), *E. sauromates* (yellow), and *E. urartica* sp. nov. (red). The species are identified based on their mtDNA haplotypes. Circle sizes are proportional to the number of specimens. Small black circles indicate missing or hypothetical haplotypes (alleles). Different alleles of a single heterozygous specimen are coded as a and b variants, while an asterisk indicates an allele of a homozygous specimen. The code numbers are same as used in Table 1.

The network analysis of nuclear genes in all three related clades, which correspond to *E. quatuorlineata*, *E. sauromates* s. s., and the new species, indicate incomplete lineage sorting in all loci with the exception of *RAG1* (Fig. 3). The new species, *E. sauromates* s. s., and *E. quatuorlineata* share *C*-*MOS* and *PRLR* haplotypes, while in *MC1R E. sauromates* s. s. and *E. qutuorlineata* have one haplotype in common. A very low haplotype diversity was detected in both *C*-*MOS* and *MC1R*. On the other hand, *PRLR* was more variable, with three haplotypes found in *E. sauromates* s. s., one of which was shared with both *E. quatuorlineata* and the new species. The largest diversity was observed in *RAG1*, in which we recovered six haplotypes. Three of them correspond with *E. sauromates* s. s., one with *E. quatuorlineata*, and two with the new species. DNA polymorphism values of all four analyzed nuclear markers are as follows: *C*-*MOS* (*h* = 2; *Hd* = 0.143; π = 0.031%; *S* = 1; *V* = 1; *Pi* = 0), *MC1R* (*h* = 2; *Hd* = 0.351; π = 0.054%; *S* = 1; *V* = 1; *Pi* = 1), *PRLR* (*h* = 3; *Hd* = 0.599; π = 0.60%; *S* = 7; *V* = 7; *Pi* = 7), *RAG1* (*h* = 6; *Hd* = 0.610; π = 0.13%; *S* = 6; *V* = 6; *Pi* = 6).

### Morphology

#### Comparison of Elaphe sauromates s. s. and the new species

For all morphological analyses, we split the snakes into two taxonomic groups corresponding to *E. sauromates* s. s. (abbreviated as ES in this and the following section) and the new species (EU; see Methods; Tables 1 and 2), and males and females were treated separately for the purpose of both descriptive statistics (Tables 4 and 5; Tables S3 and S4) and comparisons (Table S5).

**Table 4:** Morphometrics of *Elaphe sauromates* and *E. urartica* sp. nov.

Character (in mm except SVL/TL and SVL/HL)	*Elaphe sauromates*						*Elaphe urartica* sp. n.					
	Males			Females			Males			Females		
	*N*	Range	Mean ± SD	*N*	Range	Mean ± SD	*N*	Range	Mean ± SD	*N*	Range	Mean ± SD
Snout-vent length (SVL)	23	714–1,250	937±152	14	710–1,187	929 ± 160	15	650–970	795 ± 80	7	700–970	861 ± 97
Tail length (TL)	20	166–323	235 ± 42	13	135–253	196 ± 36	15	146–245	201 ± 27	7	150–235	182 ± 31
Total length	20	892–1,503	1,164 ± 191	13	845–1,439	1,115 ± 196	15	796–1,193	996 ± 104	7	850–1,205	1,043 ± 123
SVL/TL	20	3.43–5.29	3.98 ± 0.48	13	4.13–5.31	4.72 ± 0.40	15	3.46–4.45	3.99 ± 0.29	7	4.13–5.65	4.79 ± 0.20
Head length (HL)	10	24.1–34.6	29.3 ± 2.9	10	24.5–33.0	28.6 ± 3.0	7	25.4–31.0	27.9 ± 2.0	5	26.3–31.5	29.3 ± 2.4
SVL/HL	10	28.52–40.06	31.74 ± 3.18	10	28.41–36.38	32.43 ± 2.87	7	27.30–37.80	29.06 ± 3.87	5	22.30–33.92	29.61 ± 4.61
Head width (inter-ocular)	10	9.8–13.9	12.2 ± 1.2	10	10.5–15.0	12.1 ± 1.5	7	11.0–13.4	12.1 ± 1.0	5	11.0–13.1	12.1 ± 1.0
Pileus length	19	20.0–31.9	25.6 ± 3.4	12	21.0–31.6	24.0 ± 3.2	9	21.1–27.1	24.4 ± 2.0	5	23.4–27.1	25.3 ± 1.7
Pileus width	13	12.9–20.1	15.9 ± 2.1	5	12.1–17.5	13.9 ± 2.3	9	12.3–16.2	4.0 ± 1.4	5	12.5–14.8	13.5 ± 0.9
Rostrum height	13	4.0–6.9	5.6 ± 0.9	5	4.1–6.8	5.0 ± 1.1	9	3.6–6.1	4.5 ± 0.8	5	4.1–4.4	4.3 ± 0.1
Rostrum width	13	6.4–10.2	7.8 ± 1.1	5	6.1–9.4	7.3 ± 1.2	9	5.7–7.8	6.9 ± 0.8	5	6.1–8.0	7.2 ± 1.0
Inter-nostril width	13	6.3–10.5	8.3 ± 1.3	5	7.0–10.0	8.0 ± 1.2	9	5.6–8.9	7.1 ± 1.1	5	6.5–8.3	7.5 ± 0.8
Eye diameter	13	4.3–6.5	5.5 ± 0.7	5	4.7–6.1	5.4 ± 0.6	9	4.2–6.6	4.9 ± 0.7	5	4.8–5.5	4.9 ± 0.3
Supraocular plate width	13	3.6–6.9	5.0 ± 1.0	5	4.0–6.1	4.9 ± 0.8	9	3.8–5.1	4.2 ± 0.4	5	4.3–4.7	4.5 ± 0.2
Frontal plate length	13	8.1–12.5	9.2 ± 1.1	5	7.3–10.5	8.2 ± 1.3	9	6.2–8.5	7.5 ± 0.9	5	6.9–8.3	7.6 ± 0.5
Frontal plate width	13	5.6–8.6	6.8 ± 0.8	5	5.5–7.4	6.5 ± 0.7	9	5.7–6.7	6.2 ± 0.4	5	5.2–6.5	5.8 ± 0.5
Anterior inframaxillar scute length	13	7.6–14.1	10.0 ± 1.9	5	7.8–11.6	9.1 ± 1.5	9	6.7–9.7	8.0 ± 1.0	5	6.8–7.8	7.5 ± 0.4
Posterior inframaxillar scute length	13	6.5–12.5	8.8 ± 2.0	5	6.3–10.2	8.1 ± 1.7	9	5.2–8.5	6.8 ± 1.2	5	5.4–6.8	5.7 ± 0.6

**Note:**

Plates on the left side were measured.

**Table 5:** Scale counts in *E. sauromates* and *E. urartica* sp. nov.

Character	*Elaphe sauromates*						*Elaphe urartica* sp. n.					
	Males			Females			Males			Females		
	*N*	Range	Mean ± SD	*N*	Range	Mean ± SD	*N*	Range	Mean ± SD	*N*	Range	Mean ± SD
Ventrals	22	199–214	205 ± 4	11	206–222	212 ± 5	12	154–206	196 ± 14	10	194–211	204 ± 6
Subcaudal scale pairs	23	64–79	75 ± 3	15	61–72	66 ± 3	19	65–74	70 ± 3	13	60–72	64 ± 4
Dorsals and temporals touching parietals	20	9–20	13 ± 3	9	11–15	13 ± 1	12	9–14	11 ± 2	10	10–15	11 ± 1
Rows of keeled scales	10	0–19	11 ± 6	6	9–20	14 ± 4	7	18–21	19 ± 1	5	19–20	20 ± 1
Preventrals	22	0–3		11	1–2		12	1–2		10	0–3	
Rows of dorsals one head length posterior to the head	17	21–25		6	21–27		12	23–25		10	25	
Rows of dorsals at midbody	26	23–25		16	24–25		19	23–25		13	24–25	
Rows of dorsals one head length posterior to the cloaca	17	18–19		6	19–21		12	19		10	18–19	
Preoculars	23	1–3		13	1–2		19	1–3		13	1–2	
Loreals (tip of)	19	1–3		9	1–3		12	1–2		10	1–2	
Postoculars	21	1–2		11	2		19	1–2		13	2–3	
Temporals	23	1–3		15	2–3		19	2		13	2–3	
Posttemporals	23	2–5		15	2–5		19	2–4		13	3–4	
Labials	26	7–9		16	8–10		18	8		13	8–9	
Sublabials	26	9–12		15	9–12		15	10–13		11	10–12	
Gulars between posterior labials	15	10–16		6	13–15		12	13–16		10	11–16	
Gulars between anterior intermaxillars	20	0–2		10	1		12	1–2		10	1–2	
Gulars between posterior intermaxillars	20	0–4		10	1–4		12	2–4		10	2–5	

First, we asked whether both species differ in their lengths. Indeed, we found the difference between males EU and ES (SVL: *t*(36) = 3.359, *p* = 0.002, *n*(ES) = 23, *n*(EU) = 15; total length: *t*(33) = 3.082, *p* = 0.004, *n*(ES) = 20, *n*(EU) = 15), with ES males (937 ± 152 mm on average) being longer than EU males (795 ± 80 mm; Table 4) on average. Although EU females seem to be generally shorter (861 ± 97 mm) than ES females (929 ± 160 mm; Table 4), the differences are not statistically significant (SVL: *t*(19) = 0.932, *p* = 0.363, *n*(ES) = 14, *n*(EU) = 7; total length: *t*(18) = 0.794, *p* = 0.438, *n*(ES) = 13, *n*(EU) = 7). Next, we asked whether species differ in the relative lengths of their tails and heads. We found no significant differences between the species when the straight head length (Males: *F*(2,11) = 1.506, *p* = 0.264, Wilks’ Λ = 0.785, *n*(ES) = 8, *n*(EU) = 7; Females: *F*(2,10) = 1.536, *p* = 0.262, Wilks’ Λ = 0.765, *n*(ES) = 9, *n*(EU) = 5) or pileus length (Males: *F*(2,12) = 1.512, *p* = 0.260, Wilks’ Λ = 0.799, *n*(ES) = 9, *n*(EU) = 7; Females: *F*(2,10) = 3.188, *p* = 0.078, Wilks’ Λ = 0.653, *n*(ES) = 11, *n*(EU) = 5) was used as a proxy of the total head length. The between-subject effects indicated that neither tail length nor head length differed between the two species. We then analyzed the relative head size and proportions by comparing several other head dimensions with head length used as a co-variate (see Table 4; Table S2). While the full models were not significant in either sex (Males: *F*(8,1) = 2.717, *p* = 0.439, Wilks’ Λ = 0.044, *n*(ES) = 4, *n*(EU) = 7; Females: *F*(5,1) = 1.035, *p* = 0.629, Wilks’ Λ = 0.162, *n*(ES) = 3, *n*(EU) = 5), the between-subject comparisons showed that several head dimensions differed between the species (Bonferroni correction alpha = 0.0042). In males these were pileus length, rostrum height, frontal plate length, anterior inframaxillar scute length, while females only differed in pileus length. EU has relatively longer pileus, but the scutes are relatively longer in ES than in EU (see Table 4). Next, we compared counts of selected scales on the head and body. We found a difference between EU and ES males (*F*(1,23) = 3.235, *p* = 0.030, Wilks’ Λ = 0.207, *n*(ES) = 13, *n*(EU) = 12), but not between females (*F*(1,12) = 2.658, *p* = 0.449, Wilks’ Λ = 0.030, *n*(ES) = 4, *n*(EU) = 10). The between-subject comparison showed that significant differences in males were in the numbers of subcaudal scale pairs (75 ± 3 in ES; 64 ± 4 in EU) and loreal (1–3 in ES; 1–2 in EU) scales. Though not significant after Bonferroni correction, the analyses indicated possible additional differences in ventral, temporal, and gular (between the anterior intermaxillars) scales in males and ventral, loreal, and gular scales in females (Table 5). Morphological data originating from the type material of the new species can be found in Table 6 and data on a few juvenile specimens originating from Armenia in Table S4. Due to very low numbers, these were not subjected to statistical comparisons.

**Table 6:** Morphological characters of holotype and paratypes of *E. urartica* sp. nov. from Turkey (TR), Armenia (AR), and Azerbaijan (AZ).

Measure/Count	Holotype	Male 1	Male 2	Male 3	Male 4	Male 5	Male 6	Male 7	Female 1	Female 2	Female 3	Female 4	Female 5
	TR ZDEU 26/2012	TR ZDEU 114/1970	AR TuRE ES 0000003301	AR TuRE ES 0000003302	AZ IZANAS T-17	AZ IZANAS 70	AZ IZANAS 68	AZ IZANAS 518	AR TuRE ES 0000003300	AR TuRE ES 0000003303	AZ IZANAS 69	AZ IZANAS 71	AZ IZANAS 529
Snout-vent length	803	812	750	701	860	792	848	790	872	892	951	700	880
Tail length	210	229	176	177	221	213	245	202	168	158	203	150	200
Total length	1,013	1,041	926	878	108	1,005	1,093	992	1,040	1,050	1,154	850	1,080
Pileus length	24.9	26.8	22.6	21.1	24.6	25.4	27.1	24.4	23.7	23.4	25.8	27.1	26.8
Pileus width	16.2	13.9	12.6	12.3	14.6	14.9	15.7	13.0	12.9	12.5	13.3	14.1	14.8
Rostrum height	5.3	6.1	4.4	4.4	3.6	4.4	4.8	4.00	4.4	4.4	4.4	4.4	4.1
Rostrum width	6.6	7.8	5.9	5.7	6.9	7.7	7.6	7.0	6.2	6.1	8.0	8.0	8.0
Inter-nostril width	8.5	5.6	6.3	6.2	6.6	8.1	8.9	6.5	6.8	6.5	8.2	8.3	7.7
Eye diameter	4.8	4.9	4.7	4.7	4.9	6.6	5.4	4.2	4.8	4.8	4.8	5.5	4.9
Frontal plate length	8.5	8.4	6.4	6.2	8.2	7.6	8.3	6.8	7.2	6.9	7.8	7.5	8.3
Frontal plate width	6.6	6.7	5.9	5.7	6.5	6.6	6.5	5.8	5.3	5.2	6.0	5.8	6.5
Anterior inframaxillar length	8.5	7.9	8.1	7.1	8.4	9.7	9.1	7.8	7.8	5.4	7.7	7.7	7.6
Posterior inframaxillar length	6.9	6.5	6.5	5.2	8.5	7.8	8.2	6.9	6.8	5.4	5.7	5.4	6.8
Ventrals	202	195	205	204	202	199	197	154	211	206	209	194	209
Subcaudal scale pairs	72	73	67	65	68	74	70	69	60	60	70	60	63
Dorsals and temporals touching parietals	9	13	12	11	10	9	14	10	10	11	12	11	12
Preventrals	1	1	1	1	1	1	1	1	1	2	1	1	1
Rows of dorsals one head length posterior to the head	25	25	25	25	23	24	25	24	25	25	25	25	25
Rows of dorsals at midbody	23	25	25	25	23	25	23	23	25	25	25	25	25
Preoculars (left/right)	2/2	2/2	2/1	2/2	3/3	2/3	2/2	2/2	1/1	2/2	2/2	2/2	2/2
Loreals (left/right)	1/1	1/1	1/1	2/2	1/1	1/1	1/1	1/1	1/1	1/1	1/1	2/1	1/1
Postoculars (left/right)	2/2	2/2	1/2	2/2	2/2	2/2	2/2	2/2	2/2	2/2	2/2	2/2	3/3
Temporals (left/right)	2/2	2/2	2/3	2/2	2/2	2/2	2/2	2/2	2/2	2/2	3/3	2/2	3/3
Posttemporals (left/right)	4/4	2/3	4/3	4/4	4/4	4/4	4/4	4/3	4/4	4/4	3/4	3/3	4/3
Labials (left/right)	8/8	8/8	8/8	8/8	8/8	8/8	8/8	8/8	8/8	8/8	8/8	8/8	8/8
Sublabials (left/right)	11/11	12/12	13/11	10/10	11/11	10/9	11/11	11/10	11/12	11/11	10/11	11/11	10/10
Gulars between posterior labials	15	15	16	13	13	13	13	13	14	11	15	13	14

#### Sexual dimorphism

We found no differences in the length between males and females of ES (SVL: *t*(35) = 0.190, *p* = 0.850 *n*(M = Males) = 23, *n*(F = Females) = 14; total length: *t*(31) = 0.745, *p* = 0.462, *n*(M) = 20, *n*(F) = 13) and EU (SVL: *t*(20) = 1.626, *p* = 0.120, *n*(M) = 15, *n*(F) = 7; total length: *t*(20) = 0.898, *p* = 0.380, *n*(M) = 15, *n*(F) = 7), however, the sexes of both taxa differed in the relative tail lengths (ES: *F*(2,23) = 21.107, *p* = 0.000, Wilks’ Λ = 0.353, *n*(M) = 16, *n*(F) = 11; EU: *F*(2,10) = 43.678, *p* = 0.000, Wilks’ Λ = 0.103, *n*(M) = 9, *n*(F) = 5; pileus length was used as the total head length proxy in MANCOVA, SVL as a co-variate). The tails were longer in males than in females, which is also illustrated by the SVL/tail length indices–in males the tail length forms about 25% of the SVL, while it is around 21% in females (Table 4).

We also did not find overall differences in the relative head size (ES: *F*(4,1) = 1.406, *p* = 0.554, Wilks’ Λ = 0.151, *n*(M) = 4, *n*(F) = 3; EU: *F*(9,1) = 5.492, *p* = 0.320, Wilks’ Λ = 0.020, *n*(M) = 7, *n*(F) = 5), although the between-subject comparisons indicate that some differences may exist in pileus size (length and width) and eye diameter (Table 4).

There are also no sexual differences in scalation in ES (*F*(1,15) = 2.575, *p* = 0.237, Wilks’ Λ = 0.082, *n*(M) = 13, *n*(F) = 4), but according to the between-subject comparisons some differences may exist in numbers of ventral and subcaudal scales. However, model showed differences in scalation between males and females of EU (*F*(1,20) = 3.995, *p* = 0.036, Wilks’ Λ = 0.111, *n*(M) = 12, *n*(F) = 10), which was mainly driven by differences in numbers of subcaudal scale pairs (70 ± 3 in males, 64 ± 4 in females) and preocular (1–3 in males, 1–2 in females) scales. More thorough morphological comparison of larger and more complete datasets will be necessary to obtain a more robust picture of both interspecific and intersexual differences in both species.

#### Systematic account

Our findings indicate that the taxon *E. sauromates* s. l. (type locality of *E. sauromates*: Pre-Sivash area of the Crimean peninsula, the Perekop Isthmus and adjacent territories of the Lower Dnieper region; related sample nos. 1,178 and 1,179 in this study; see Table 1) is composed of two clearly genetically differentiated allopatric populations. The populations from Transcaucasia and eastern Anatolia, which are also morphologically differentiated from *E. sauromates* s. s., represent a cryptic phylogenetic lineage. In accordance with the definition of the genetic species concept (i.e., genetic species is a group of genetically compatible interbreeding natural populations that is genetically isolated from other such groups; Baker & Bradley, 2006) and evolutionary species concept (i.e., populations with a long independent evolutionary history, represents a lineage of ancestral descendent populations, and maintains its identity from other lineages both on genetic (mtDNA and nDNA) and morphological level; Simpson, 1951; Wiley, 1978), we describe this lineage as a new species:

Family Colubridae
Genus: *Elaphe* Fitzinger in Wagler, 1833
*Elaphe urartica* Jablonski, Kukushkin, Avcı, Bunyatova, Ilgaz, Tuniyev et Jandzik sp. nov.
urn:lsid:zoobank.org:act:A8F964EB–FD8E–4EAB–9549–DECD0648075C

*Coluber* sp. (No. 15)—Hohenacker, 1831: 374; *Coluber* sp. (No. 18)—Hohenacker, 1831: 375; *C. cereus*—Dwigubsky, 1832: 27, (*nomen dubium*); *C. fulvus*—Dwigubsky, 1832: 28, (*nomen dubium*); *C. taeniothys* Fischer von Waldheim, (*nomen dubium*), 1832: 575; Hohenacker, 1837: 145; *Tropidonotus sauromates* Eichwald, 1841: 140; *Elaphis sauromates* Duméril, 1853: 453 (part.); Strauch, 1873: 92 (part.); *C. quatuorlineata sauromates* Boulenger, 1894: 47 (part.); *C. dione* var. *sauromates* Nikolsky, 1905: 257 (part.); *C. quatuorlineata* var. *sauromates* Schreiber, 1912: 698 (part.); *E. quatuorlineata* Nikolsky, 1916: 133 (part.); Alekperov, 1978: 128; *E. quatuorlineata sauromates* Lindholm, 1929: 80 (part.); Sobolevssky, 1929; Szczerbak, 1966: 200 (part.); Muskhelishvili, 1970: 163; Schulz, 1996: 224 (part.); Sindaco et al., 2000: 473 (part.); *E. sauromates* Lenk, Joger & Wink, 2001: 329 (part.); Utiger et al., 2002: 105 (part.); Tuniyev et al., 2009: 80 (part.); Arakelyan et al., 2011: 88; Kornilios et al., 2014: 149 (part.); Wallach, Williams & Boundy, 2014: 263 (part.); Safaei-Mahroo et al., 2015: 279. For further details on nomenclature, see the Supplementary Information.

**Figure 4:**
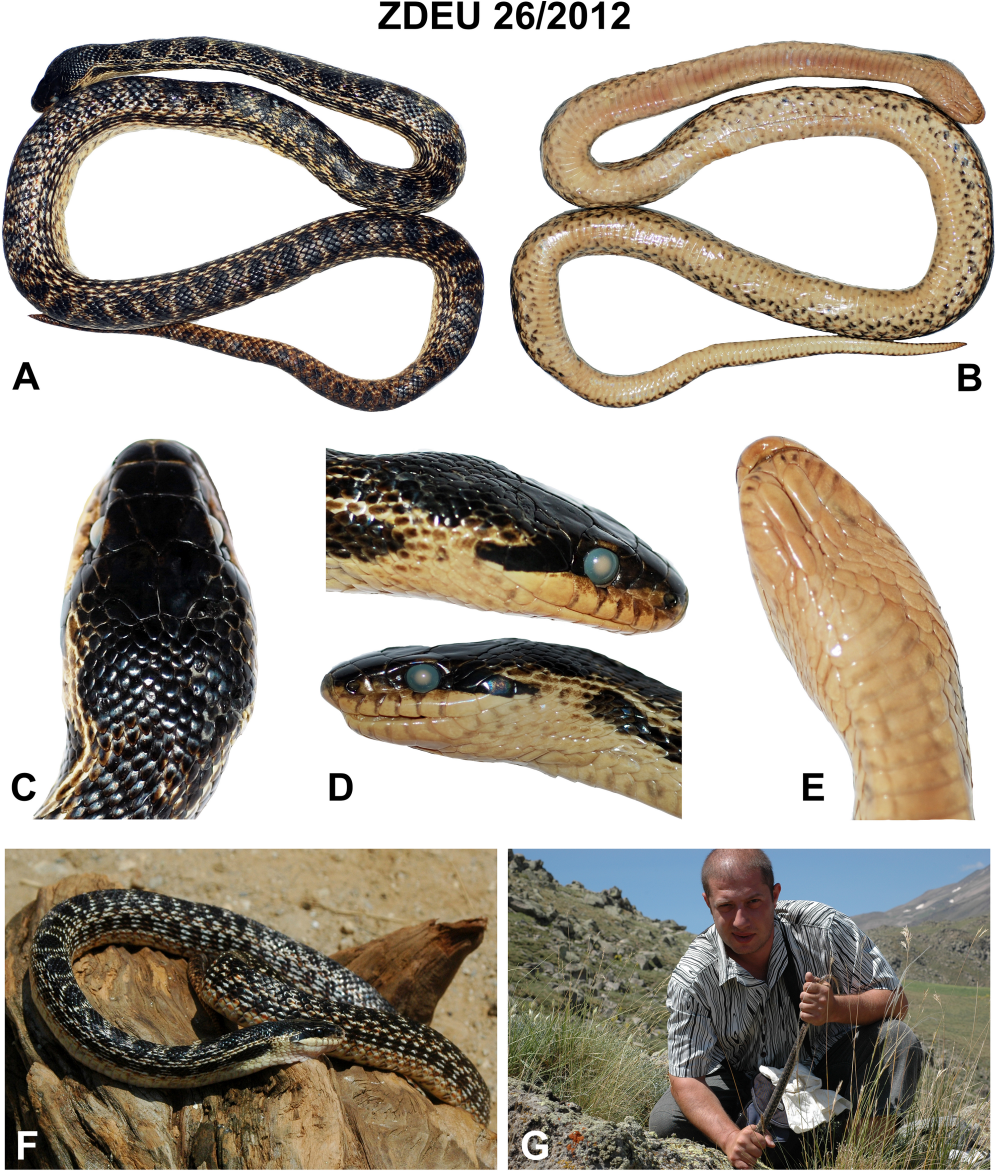
Holotype (ZDEU 26/2012) of *Elaphe urartica* sp. nov. from eastern Turkey. (A) Dorsal view, (B) ventral view, (C) dorsal view of the head, (D) lateral view, (E) ventral view (photos by Aziz Avcı), (F) the holotype while alive (photo by Çetin Ilgaz), (G) Sako B. Tuniyev with freshly caught holotype of *E. urartica* sp. nov. (photo by Boris Tuniyev).

**Figure 5:**
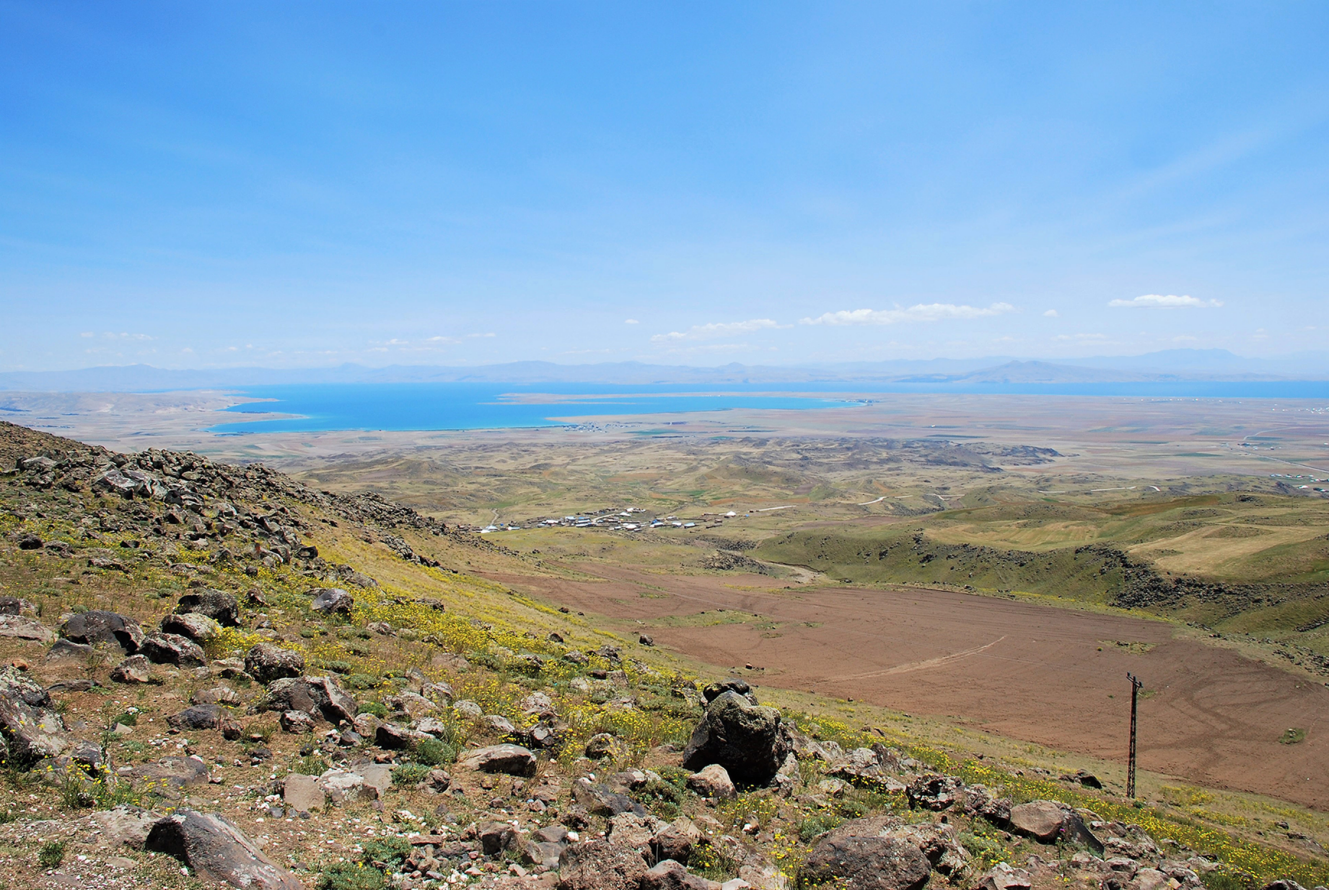
Habitat at the type locality (Kısıklı, Süphan Mts., Turkey) of *Elaphe urartica* sp. nov. in south-eastern Turkey (photo by Boris Tuniyev).

*Holotype*. (Fig. 4). ZDEU 26/2012 (tissue sample no. 1,124), adult male from Bitlis Province, Turkey (Kısıklı Village, Süphan Mts.; 38.93°N, 42.91°E, 2,394 m a. s. l.; Fig. 5), collected by Sako B. Tuniyev; July 16, 2012 (see also Tuniyev et al., 2014).

**Figure 6:**
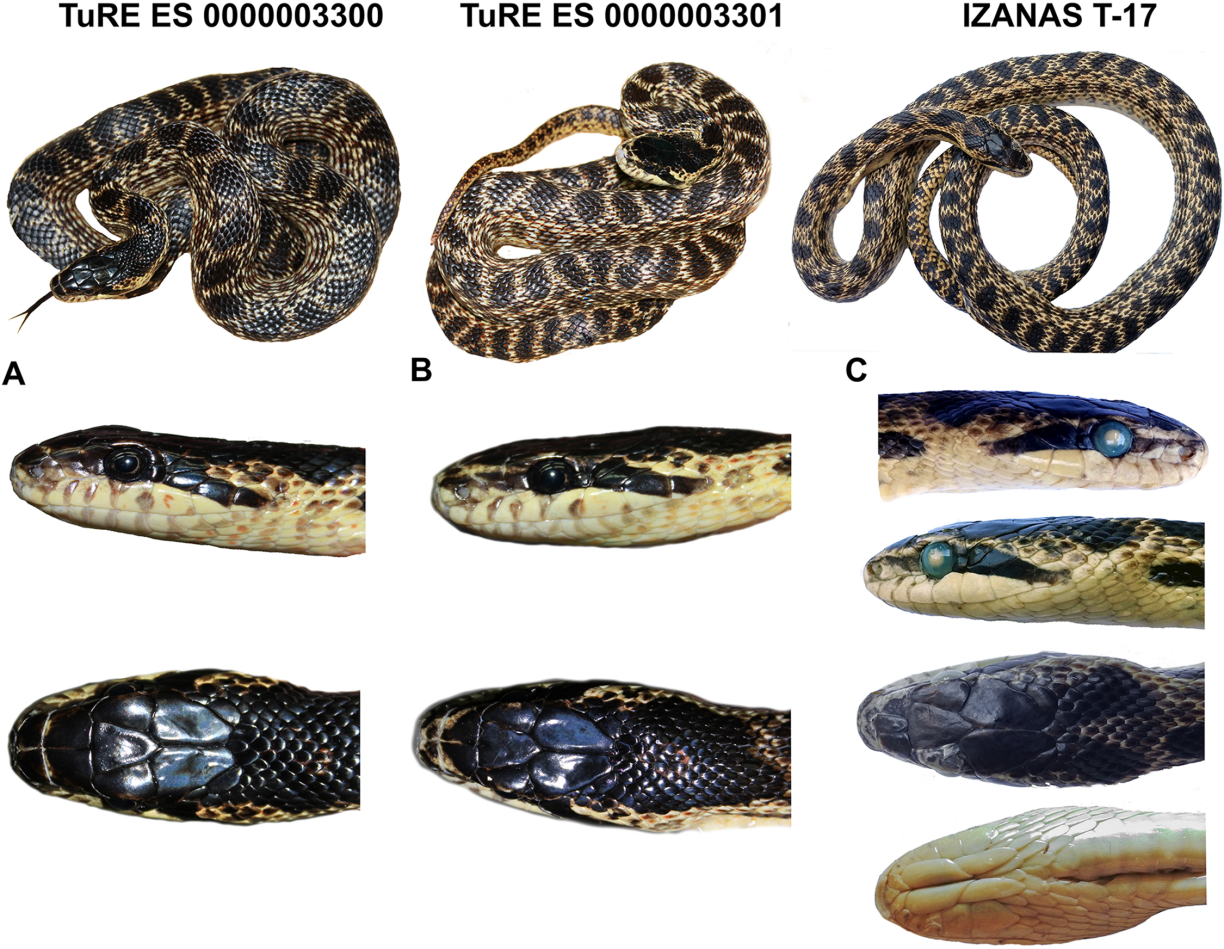
Paratypes of *Elaphe urartica* sp. nov. from Armenia ((A and B) photo by Ilya Korshunov and Konstantin Shiryaev) and Azerbaijan ((C) photo by Sabina Bunyatova) showing the habitus and details of the head. For the locality details, see Table 1.

*Paratypes*. (Fig. 6; Table 6). 12 specimens (seven males, five females; eight fixed specimens and four alive).

**Figure 7:**
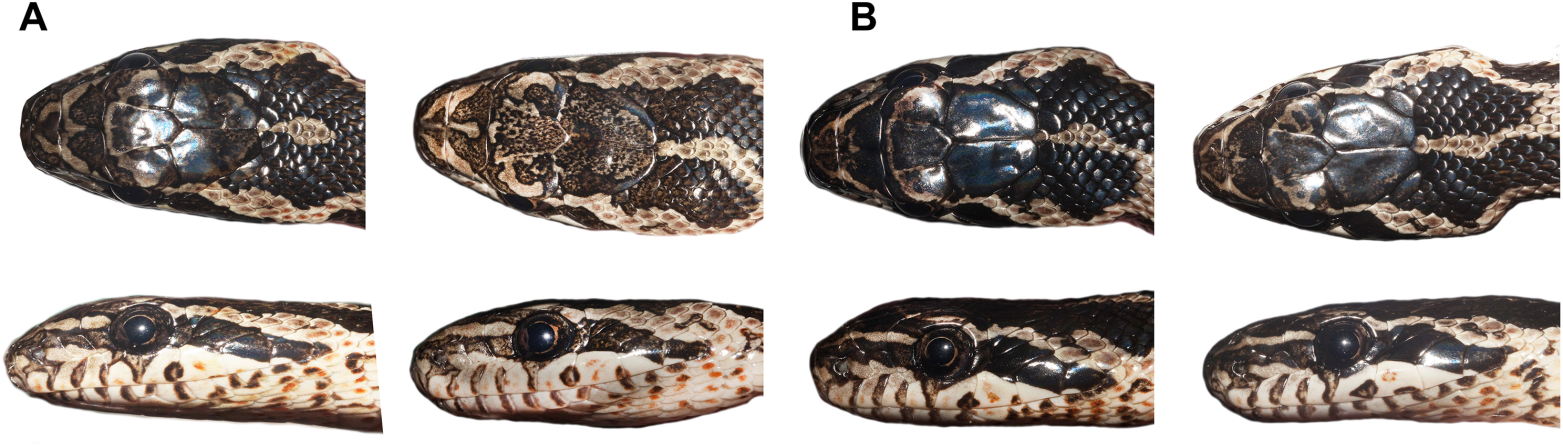
Head coloration of juvenile *Elaphe urartica* sp. nov. from Armenia. (A) Males, (B) females (photos by Ilya Korshunov and Konstantin Shiryaev).

ZDEU 114/1970, adult male from Kars Province (Hoşerenler Plate), Turkey, 40.61°N, 43.09°E; IZANAS T-17, (tissue sample 3,655) adult male from surroundings of Guzdak, Qobustan, Azerbaijan, 40.37°N, 49.68°E (Fig. 6C); IZANAS 518, adult male from surroundings of Zaqatala, Azerbaijan, 41.63°N, 46.65°E; IZANAS 529, adult female from surroundings of Zaqatala, Azerbaijan, 41.63°N, 46.65°E; IZANAS 68, adult male from surroundings of Lenkoran, Azerbaijan, 38.75°N, 48.85°E; IZANAS 71, adult female from Zivi-Zkaro (=Tsivi-Tskaro; now Soyuqbulaq), Azerbaijan, 41.32°N, 45.27°E; IZANAS 70, adult male from surroundings of Şamaxi, Azerbaijan, 40.63°N, 48.63°E; IZANAS 69, adult female from Şamaxi, Azerbaijan; TuRE ES 0000003300 (tissue sample 3,196), live adult female from Mt. Gutanasar, Gegamsky Ridge (=Ahmangan), near Abovyan, Armenia, 40.37°N, 44.69°E (Fig. 6A); TuRE ES 0000003301 (tissue sample 3,195), live adult male from Mt. Gutanasar, Gegamsky Ridge (=Ahmangan), near Abovyan, Armenia, 40.37°N, 44.69°E (Fig. 6B); TuRE ES 0000003302 (tissue sample 3,450), live adult male from surroundings of Mt. Atis, Kotayikskoe Plateau, Armenia, 40.36°N, 44.61°E; TuRE ES 0000003303 (tissue sample 3,449), live adult female from NW slope of the Mt. Atis, Kotayikskoe Plateau, Armenia, 40.31°N, 44.73°E.

*Etymology*. The specific epithet is a feminine adjective derived from the name of the ancient kingdom of Urartu that flourished in the Armenian Highlands and around lake Van, an area of recent distribution of *E. urartica* sp. nov., in the 9th–6th century BCE (Asher & Asher, 2009). We are choosing this name out of respect for Peter Simon Pallas, who proposed the name for *E. sauromates*, now the sister species of *E. urartica*, which most likely refers to Sarmatians (Sauromatae; *Σαυρομαται* in Greek), a confederation of nomadic peoples inhabiting vast portions of the recent range of *E. sauromates* between the 5th century BCE and 4th century CE.

*Diagnosis*. A new species of western Palearctic genus *Elaphe*, very similar to *E. sauromates* (Pallas, 1814), characterized by the combination of the following characters: total length usually does not exceed 1,200 mm (796–1,205 mm), snout-vent (SVL) length usually less than 1,000 mm (650–970 mm), tail length less than 250 mm (146–245 mm) (see Tables 4 and 6). Tail forms about 25% of the SVL in males and about 21% in females. Head relatively large, distinguished from the body. Snout in prefrontal and internasal area is conspicuously convex which usually forms a hook-nosed head profile. Pileus length on average 1.8–1.9 times larger than its width. Frontal plate 1.2–1.3 times longer than wide. Anterior inframaxillar scute relatively large and wide, 1.2–1.3 times longer than the narrow posterior inframaxillar scute. One or two preocular scales, one loreal, two postoculars, two temporals, three or four posttemporals, eight labials, 10–11 sublabials on each side of the head. Eye in contact with fourth and fifth labials (Table 5; Table S3). Variation in head scale counts is relatively low (see Table S3). Usually two gulars located the anterior inframaxillars. The total number of gulars between inframaxillars and first preventral scale exceeds 12. Number of ventrals is 154–211 (154–206 in males, 194–211 in females), 60–74 subcaudal pairs (65–74 in males, 60–72 in females). 23–25 longitudinal rows of scales are around the midbody, with well-developed keels on 18–21 rows of body scales. The background of dorsal surfaces of the body and lateral surfaces of the head are yellowish or whitish, or seldomly bright yellow. The pattern of the dorsal surface of the body is composed of 50–65 rounded brown or black large ellipsoid spots, which may have whitish edges. Spots can be extended transversely in the posterior part of the body. Pileus is dark, often almost black, slightly lighter on the tip of the snout. Upper preoculars and temporals are dark forming a postocular stripe extending toward the mouth corner. This stripe blends with the dark dorsolateral head coloration anterior to the eye. Pale spots on the labials, only barely visible or lacking on sublabials. Ventral side of the body is whitish to pale yellow, sometimes with pinkish tint. There are marbled patterns of numerous small irregular dark brown and light gray spots with reddish contours that are more pronounced on the lateral sides of ventral plates. Throat is light, with numerous reddish-orange or brownish speckles on the lower jaws and anterior ventral plates. Iris is dark brown or almost black with a thin light rim around the pupil.

**Figure 8:**
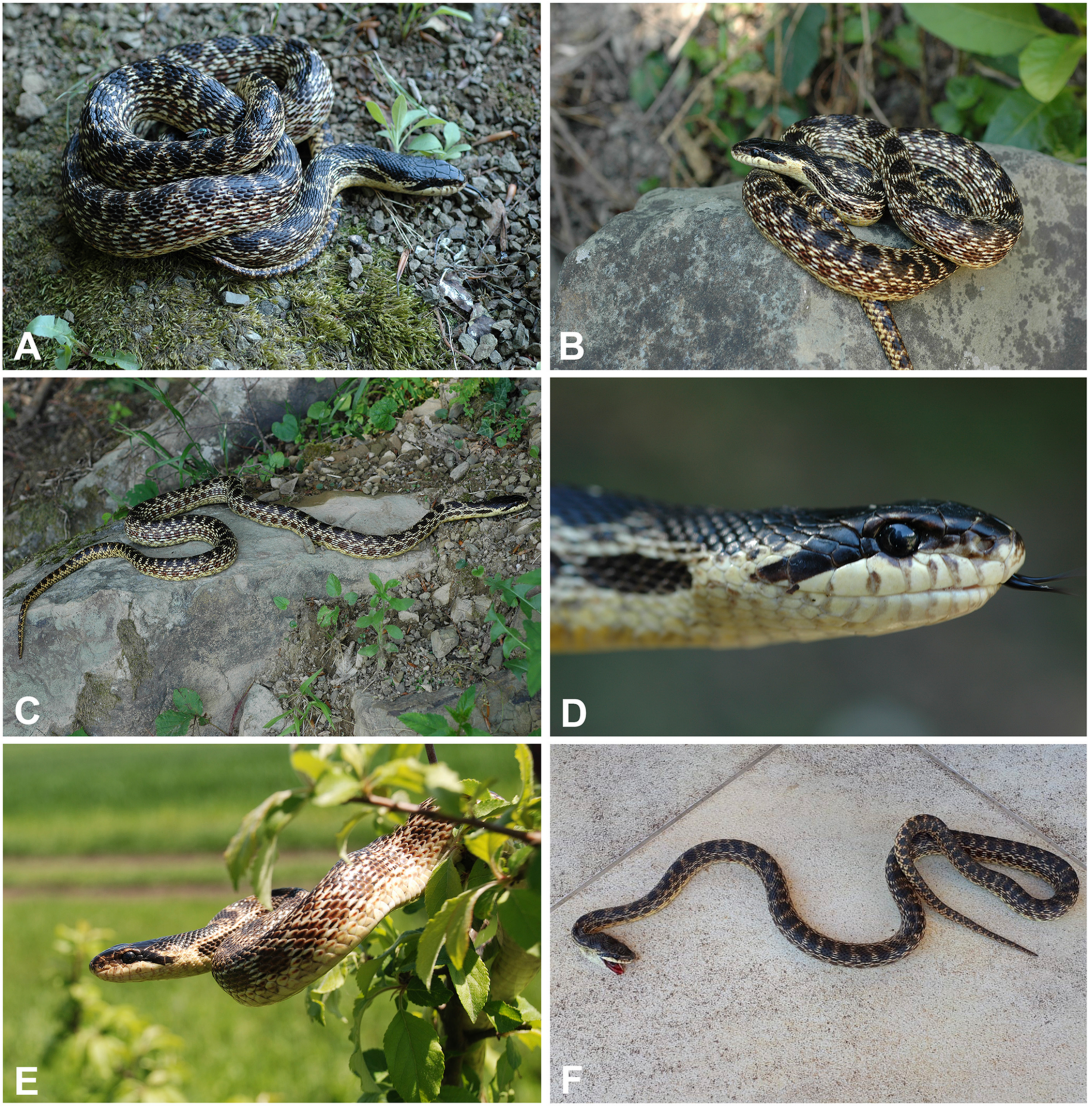
Color and pattern variation in *Elaphe urartica* sp. nov. (A–D) Kaputan, Armenia; (E) Didi Shiraki, Georgia; (F) Ersi, Dagestan, Russian Federation (photos by Boris Tuniyev).

**Figure 9:**
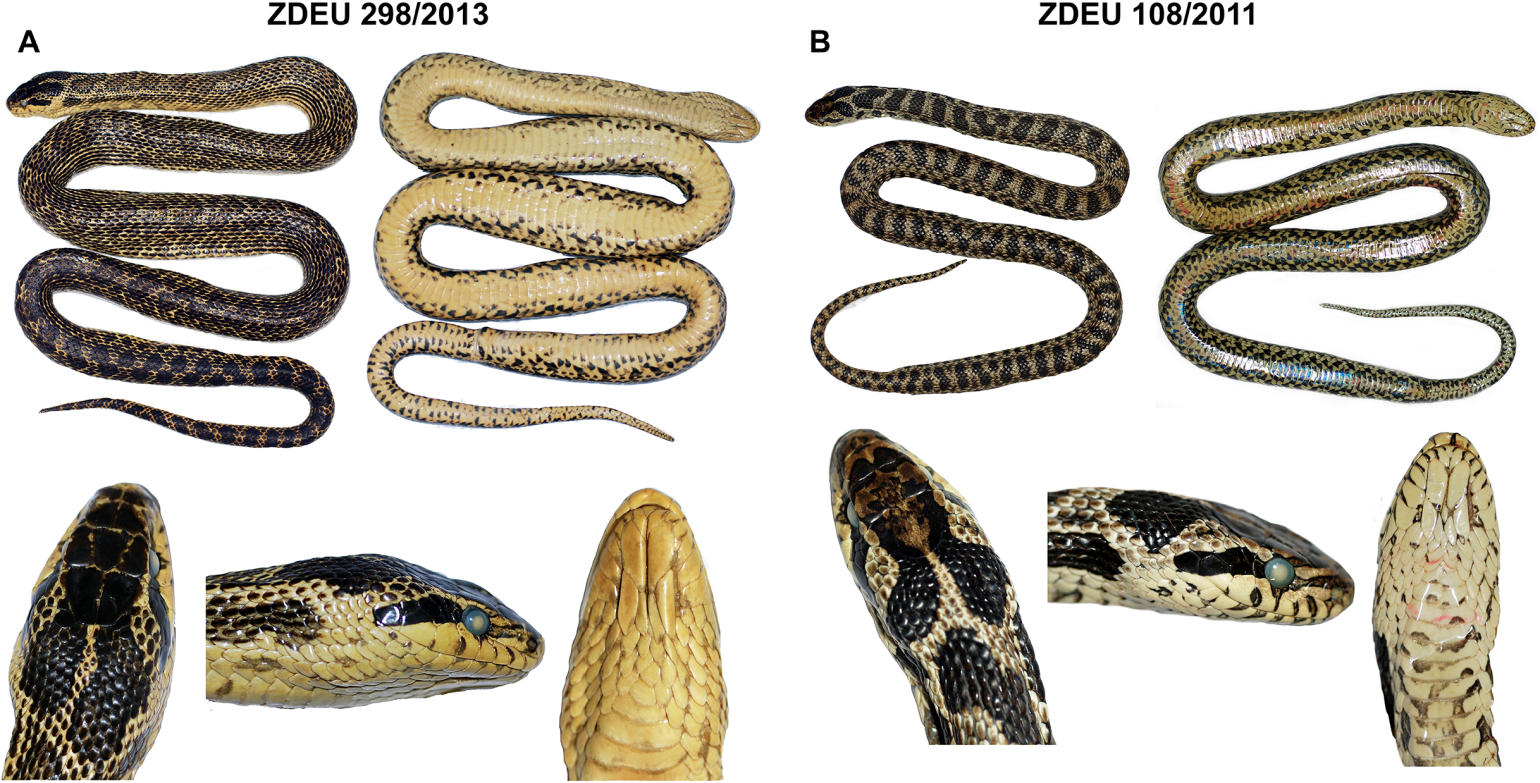
Coloration and pattern of adult (A) and subadult (B) males of *Elaphe sauromates* from south-western Turkey (photos by Aziz Avcı). For the locality details, see Table 1.

**Figure 10:**
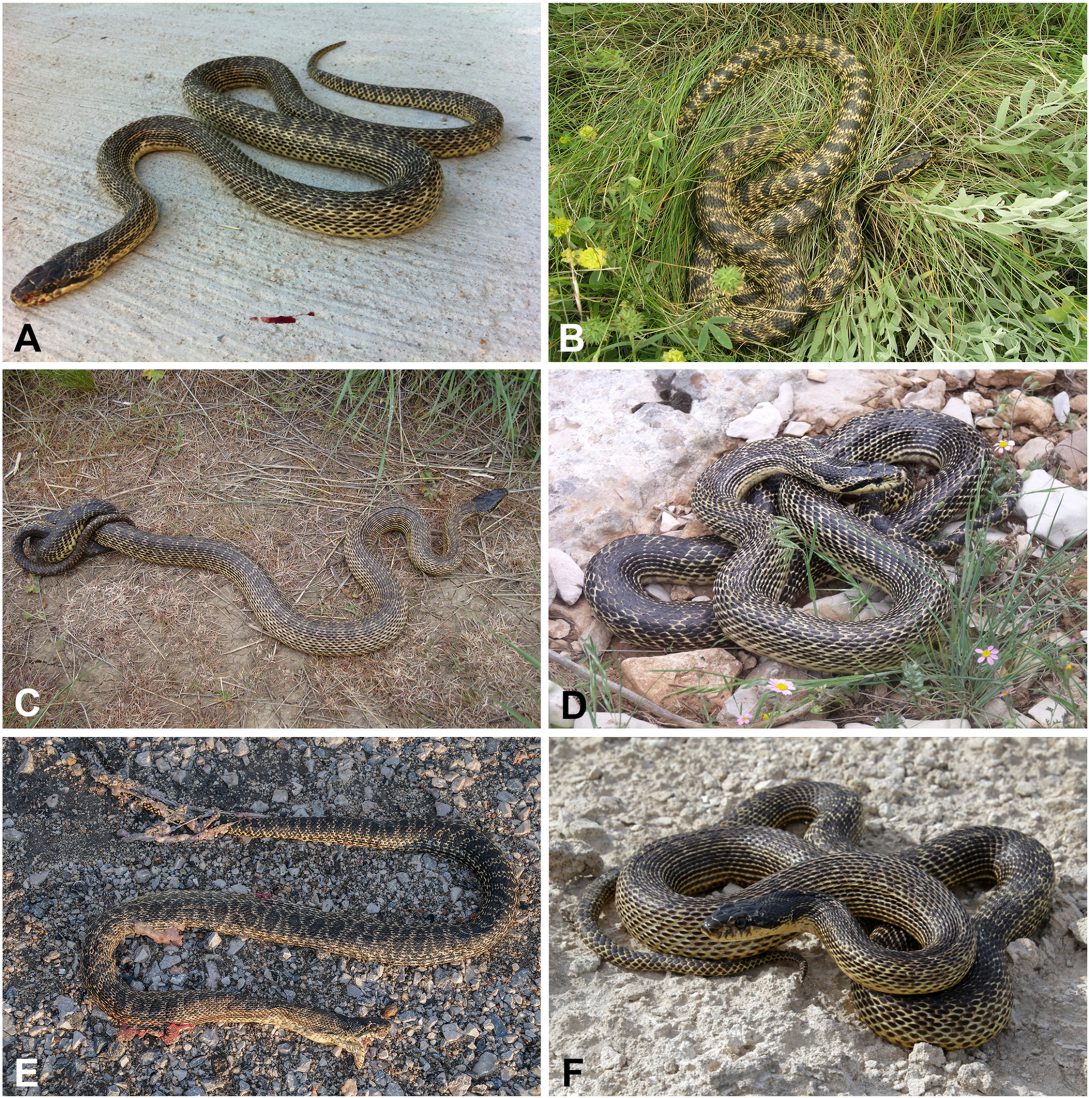
Color and pattern variation in *Elaphe sauromates*. (A) Gelendzhik, Krasnodar Territory, Russian Federation (photo by Boris Tuniyev); (B) Chauda Cape, Crimea (photo by Egor Kalmykov & Oleg Kukushkin); (C) Solenoe Ozero, Crimea (photo by Anton Nadolnyi); (D) Göltarla, Antalya, Turkey (ZDEU 298/2013); (E) Iskenderun, Turkey (photo by Daniel Jablonski), (F) Ustyurt, Kazakhstan (photo by Mark Pestov).

*Differential diagnosis. Elaphe urartica* sp. nov. is closely related to *E. sauromates* and *E. quatuorlineata*. The genetic distance between *E. urartica* sp. nov. and *E. sauromates* is 7.20% and 6.91% in *COI* and *ND4*, respectively, and 0.15%, 0.15%, 0.73%, and 0.24%, in *C-MOS*, *MC1R*, *PRLR*, and *RAG1*, respectively. The genetic distance between *E. urartica* sp. nov. and *E. quatuorlineata* is 8.24% and 5.80% in *COI* and *ND4*, respectively, and 0.15%, 0.15%, and 0.20%, in *C-MOS*, *MC1R*, and *RAG1*, respectively. *E. urartica* sp. nov. is also morphologically very similar to *E. sauromates. E. urartica* sp. nov. attains shorter lengths than *E. sauromates* (795 ± 80 mm vs. 937 ± 152 mm in *E. sauromates* males, 861 ± 97 mm vs. 929 ± 160 mm in *E. sauromates* females; though *E. sauromates* from other parts of the range, for example, the Balkans, can be even larger than specimens in our dataset, see Sahlean et al., 2016). Both taxa also differ in some relative head dimensions. *E. urartica* sp. nov males have relatively (in comparison to the head length) longer pileus, higher rostrum, but shorter frontal plate and anterior inframaxillary scute. Females only differ in the pileus length (Table 4). The upper surface of the head is more convex near orbits, prefrontals, and internasals and the rostrum is more anteriorly pronounced in *E. urartica* sp. nov. than in *E. sauromates*. Males of both species also slightly differ in their scalation: *E. urartica* sp. nov. males have fewer subcaudal pairs (64 ± 4 vs. 75 ± 3 in *E. sauromates*) and loreal scales (1–2 vs. 1–3 in *E. sauromates*). Other differences in metric and meristic characters were not found statistically significant. The coloration of *E. urartica* sp. nov. is generally darker than that of *E. sauromates* (Figs. 4 and 6–10). The dorsal side of the head is very dark, sometimes almost black and without the whitish area separating two blotches just posterior to the head as seen in *E. sauromates* (Figs. 4 and 6–10). On the lateral side of the head the dark stripe running from behind the eye toward the corner of the mouth is also less distinguished in *E. urartica* sp. nov. compared to *E. sauromates*, in which it is clearly separated by lighter color from the darker head coloration. *E. urartica* sp. nov. has more conspicuous dorsal body spots that are more rounded than in *E. sauromates*, in which transverse elongation of the spots is common. These dorsal spots are typically lined with whitish color in *E. urartica* sp. nov. rather than yellow or yellowish as in *E. sauromates*.

*Description of the holotype*. Adult male (ZDEU 26/2012; Fig. 4). Body cylindrical, snout-vent length 803 mm, tail length 210.00 mm. Head big and clearly distinct from the neck, pileus length 24.90 mm, width 16.20 mm. Head and body scales keeled. Rostral slightly curved toward the top of the head, indistinctly wedged between the internasals. Rostrum height 5.3 mm and width 6.6 mm, in contact with two labials, two nasals, and two internasals. Nostrils located within the nasal scales, inter-nostril width is 8.5 mm. Loreal on either side of the head in contact with second and third labials. Two preocular and postocular plates on each side of the head. Eyes circular with circular pupil of 4.8 mm diameter. Length of the narrow frontal 8.5 mm, width 6.6 mm. Eight labials, with fourth and fifth in contact with the eyes on each side. A total of 11 sublabials on each side, five sublabials in contact with the anterior chin shields on each side. Two temporals and two posttemporals on each side. Nine dorsal and temporal scales surrounding the posterior margin of the parietals. Two gular scales in contact with the anterior chin shield. A total of 23 dorsal scale rows at midbody, 25 at the level of one head length posterior to the head, and 19 at one head length anterior to the cloaca level. One preventral, 202 ventral plates, two anal plates, and 72 and 73 subcaudals on each side, respectively (Table 4).

The dorsal side of the body is yellowish with round black spots. Flanks with two rows of dark blotches. Dorsal and lateral spots form striped pattern on the tail. The top of the head is black. Temporal stripe distinct, blending with the dark dorsolateral head coloration anterior to the eye. The ventral side of the body is yellowish white, with black markings (Fig. 4).

*Variation*. Details on variation among the type specimens of *E. urartica* sp. nov. are presented in Table 6. The coloration of the paratypes is very similar to that of the holotype.

*Distribution and habitat*. The geographic range of *E. urartica* sp. nov. is bordered by the Armenian Plateau, south-eastern foothills of the Great Caucasus, Alazan Valley, Kur-Aras, Lenkoran Lowlands, and the area of Qobustan. The species is distributed in Turkey, Georgia, Armenia, Azerbaijan, Nagorno-Karabakh, Iran, and Russia. In Turkey, it can be found east of the Anatolian Diagonal with reliable records from Kars, Bitlis, Diyarbakır, and Van Provinces, presumably also in Erzurum, Iğdır, and Ağrı Provinces (Baran et al., 2012). In eastern Transcaucasia *E. urartica* sp. nov. is distributed from south-eastern Georgia to the Zalka Plateau or to Suramskyi Ridge in Southern Ossetia in the West, throughout most of the Armenian territory, Nagorno-Karabakh, and Azerbaijan with the exception of the Abşeron Peninsula. The eastern part of the range lies in northern Iran to the Golestan Province to the East, and Kermanshah and Semnan Provinces to the South (Alekperov & Loginov, 1953; Muskhelishvili, 1970; Flärdh, 1983; Schulz, 1996; Sindaco et al., 2000; Arakelyan et al., 2011; Bunyatova, Akhmedov & Dzhafarov, 2012; Bunyatova, 2013; Najafov, Hashimov & Isgenderov, 2013; Safaei-Mahroo et al., 2015). In the Russian Federation, *E. urartica* sp. nov. occurs in Samur-Devichi Lowlands of southern Dagestan and probably in the Dagestan Intermontane Region as well (Ananjeva et al., 2006; Mazanaeva & Askenderov, 2014). The species could also occur in the extreme northern regions of Iraq (Sindaco, Venchi & Grieco, 2013).

The snake occurs in a wide range of altitudes—from ca. 25 m below sea level in the Lenkoran foredeep to about 2,600 m a.s.l. in the Shirak Province in Armenia (Arakelyan et al., 2011). It is an eurytopic species inhabiting a wide variety of landscapes: mountain and lowland semi deserts, different types of the steppe, semi subtropical savannah-like forest-steppes with oreoxerophytes, sparse juniper forests, montane broad-leaved forests, and alpine meadows (Fig. 5). The climate within the *E. urartica* sp. nov. range varies from the subtropical in Lenkoran and piedmont area of eastern Transcaucasia to cold mountain climate in Armenia and north-eastern Anatolia. Humidity varies from highly arid (with annual precipitation of less than 200 mm) to moderately humid (1,400–1,600 mm per year; Clark & Clark, 1973; Arakelyan et al., 2011; Bunyatova, Akhmedov & Dzhafarov, 2012; Şensoy et al., 2016).

*Elaphe urartica* sp. nov. is sympatric with *E. dione* in Dagestan, central-eastern Azerbaijan, eastern Georgia, and presumably in north-eastern Turkey, southern Armenia, and northern Iran. All other species of the genus *Elaphe* have allopatric distribution relative to *E. urartica* sp. nov. Since the species occurs in a region of southern Russia (Dagestan), north of the Caucasus, that is geographically and politically considered a part of Europe (Sillero et al., 2014), *E. urartica* sp. nov. is considered another member of the European herpetofauna.

*Conservation status. Elaphe sauromates* is a species with declining population numbers and is listed (as *E. quatuorlineata*) in the Red Data Books of: Ukraine (1994)—category 3, Kazakhstan (1996)—category 4—a little-known species, and Turkmenistan (1999)—category 3—a rare species on the periphery of the distribution range. It is also listed among the taxa recommended to be included in the new edition of the Red Data Book of the Russian Federation as vulnerable species (requiring priority of protection measures—III) (Ananjeva et al., 2006; Iljashenko et al., 2018). The current global IUCN Red List status for *E. sauromates* s.l. is of Least Concern (Aghasyan et al., 2017). Since *E. urartica* sp. nov. occurs in the parts of the territory from which the data for these lists was derived, similar conservation concerns might apply for it as well. However, the consequence of splitting *E. sauromates* s. l. into two separate species is that the ranges of both species have significantly decreased, and this should be considered in future conservation measures as well.

*Proposal of common names*. We propose the English name “Urartian Rat Snake” for *E. urartica* sp. nov. Along with the name “Blotched rat snake”, we also suggest using the name “Sarmatian Rat Snake” for *E. sauromates*, instead of the older “Eastern Four-lined Rat Snake” derived as a subspecific name from the common name of *E. quatuorlineata*. The newly proposed name would decrease confusion and also better reflects the scientific name of *E. sauromates*.

## Discussion

### Systematics of *Elaphe sauromates* and *Elaphe urartica* sp. nov

Since the publication of Boulenger’s Catalogue of the snakes in the British Museum (1894) *E. sauromates* had been considered an eastern subspecies of *E. quatuorlineata*. Based solely on genetic distances between the two taxa, Helfenberger (2001) and Lenk, Joger & Wink (2001) proposed their split into two sister species, which has been largely accepted. The distribution of both species is parapatric (Sindaco, Venchi & Grieco, 2013; Kornilios et al., 2014). While *E. quatuorlineata* and *E. sauromates* are clearly and easily distinguishable, mainly based on adult pattern and coloration (Böhme & Ščerbak, 1993; Schulz, 1996), *E. urartica* sp. nov. represents a cryptic species, only moderately phenotypically differentiated from *E. sauromates*. Based on the available evidence, both species currently have allopatric ranges, presumably separated by the Anatolian Diagonal and the Great Caucasus (Fig. 1; see Schulz, 1996; Sindaco et al., 2000). Phylogenetically *E. urartica* sp. nov. and *E. sauromates* represent sister lineages, and together they form a lineage sister to *E. quatuorlineata* (Fig. 2). Relatively little is known about intraspecific variation of *E. urartica* sp. nov. but haplotype diversity indicates that it is much lower than in *E. sauromates* (Figs. 1 and 3; Table 3, Results). The species occurs in a relatively small range (Fig. 1), however, in the region known as a radiation and speciation center (Tuniyev, 1995). While it does not seem likely that *E. urartica* sp. nov. is further differentiated into the subspecies in eastern Anatolia or Transcaucasia, the Caucasian montane part of the range of *E. sauromates* s. l. has yet to be genetically analyzed, and thus it remains unclear what species inhabits this region. Using phenotypic identification (i.e., without genetic analysis), it seems that both species come into possible parapatry in Dagestan—with *E. sauromates* in northern plains and *E. urartica* sp. nov. in more southerly located montane habitats (B.S. Tuniyev, 2018, personal observation). *E. sauromates* has been found relatively close to the southern ranges of the Anatolian Diagonal (Kayseri Province, Turkey, sample 2,892, see Table 1; Fig. 1A). On the other hand, the Anatolian Diagonal has formed a natural barrier to this species’ ability for dispersal southward from western and central Anatolia (Davis, 1971; Nilson, Andrén & Flärdh, 1990; Jandzik et al., 2018), so it is more likely that the Levantine populations actually belong to *E. urartica* sp. nov. Given the commonly observed biogeographic pattern in this region (Jandzik, Avcı & Gvoždík, 2013; Jandzik et al., 2018; Stümpel et al., 2016; Tamar et al., 2016; Kornilios, 2017), it is also possible that these, likely, isolated populations have diverged from one of these species (or their common ancestor) and represent a yet undiscovered taxon (Zinner, 1972).

### Differentiation of *E. sauromates* and *E. urartica* sp. nov

*Elaphe urartica* sp. nov. is clearly genetically differentiated from *E. sauromates* and both taxa form statistically highly supported lineages (Fig. 2) in mtDNA trees. The uncorrected *p*-distances between both lineages in mtDNA sequences reach values around 7% (Table 3), which is very similar to distances observed in many other closely related snake species, for example, *E. dione* and *E. bimaculata* (~9%)*, E. quadrivirgata* and *E. carinata* (~8.6%), *E. quadrivirgata* and *E. schrenckii* (~8.2%), *Z. persicus* Werner, 1913 and *Z. longissimus* (Laurenti, 1768) (~8%), *Vipera ursinii* (Bonaparte, 1835), *V. graeca* Nilson & Andrén, 1988, and *V. renardi* (Christoph, 1861) (~4.5%), *Rhynchocalamus melanocephalus* (Jan, 1862) and *R. dayanae* Tamar, Šmíd, Göçmen, Meiri, Carranza, 2016 (4–10.2%) (Utiger et al., 2002; Gvoždík et al., 2012; Huang et al., 2012; Tamar et al., 2016; Mizsei et al., 2017). Two out of four nuclear genes (*MC1R* and *RAG1*) are also clearly differentiated, though closely related between *E. urartica* sp. nov. and *E. sauromates*, while the remaining two (*C-MOS* and *PRLR*) show signs of incomplete lineage sorting. Interestingly, *E. sauromates* and *E. quatuorlineata* share alleles in three of the analyzed genes, and only haplotypes of *RAG1* are mutually exclusive for each of them (Fig. 3). Such pattern of differences between mtDNA and nDNA has been observed among other closely related reptile species as well (see above or Gvoždík et al., 2010; Tamar et al., 2016; Mizsei et al., 2017) and it is to be expected as the autosomal loci have fourfold slower rate of lineage sorting (Avise, 2000).

*Elaphe urartica* sp. nov. represents a cryptic taxon within *E. sauromates* s. l. While we found some phenotypic differences, mainly being relatively smaller in size, having a moderate differences in the shape of the head, some head scalation dimensions, and rather different adult coloration, both species are still morphologically very similar (see Figs. 4 and 6–8 for *E. urartica* sp. nov. and 9, 10 for *E. sauromates*), especially when compared to their sister taxon *E. quatuorlineata*. However, this is also not unusual among closely related snake species, even within the genus *Elaphe*—*E. dione*, *E. bimaculata*, and *E. zoigeensis* Huang, Ding, Burbrink, Yang, Huang, Ling, Chen & Zhang, 2012 are virtually indistinguishable based on external morphology (Schulz, 1996; Huang et al., 2012). This similarity could be the result of similar environmental conditions and, consequently, the absence of selective pressure toward other type of coloration, size, or proportions (Fišer, Robinson & Malard, 2018). Though none of the morphological characters seem to be species-specific, using their combination will allow for confident specific identification. More robust sampling for morphological analyses will shed additional light on the levels of differentiation between both species.

### Historical biogeography

The fossil record indicates that the ancestors of modern *E. sauromates* s. l. were present in the region of the recent range since at least Late Pliocene. Snake remnants identified as either *E. quatuorlineata* or *E*. cf. *quatuorlineata*, presumably belonging to *E. sauromates* s. l., are known from the Early-Middle Pleistocene of the Balkans, Transcaucasia, and central Anatolia (Szyndlar, 1984, 1991; Venczel & Şen, 1994; Venczel, 2000; Venczel & Várdai, 2000; Kornilios et al., 2014; Wallach, Williams & Boundy, 2014; Blain et al., 2014; Blain, 2016). In addition, *Elaphe* aff. *quatuorlineata* fossils were also described from the Middle Pliocene of Moldova (Redcozubov, 1991, 2005), indicating that parts of the range were probably inhabited even earlier.

Molecular-phylogenetic analyses suggest that *E. sauromates* s. l. and *E. quatuorlineata* split from their last common ancestor sometime in the Late Miocene (7.3–8.3 Mya), presumably during the formation of the mid-Aegean Trench that separated the Balkans from Asia Minor (Lymberakis & Poulakakis, 2010; Kornilios et al., 2014). Further evolution of *E. sauromates* s. l. is mainly associated with Anatolia and adjacent areas of the southern Caucasus. The mtDNA genetic distances between *E. sauromates* s. s. and *E. urartica* sp. nov. (Table 3) place the split between these two lineages at the Miocene-Pliocene boundary (five to eight Mya; see Kornilios et al., 2014), so they presumably separated from each other not very long after their ancestors separated from *E. quatuorlineata*. Although our estimation is only approximate, it coincides with the splits among other snake and lizard taxa from the same region, most notably the montane vipers of *Montivipera xanthina* complex—*M. xanthina* clade from western Anatolia and *M. bornmuelleri* clade from the east and south of the Anatolian Diagonal separated around five to six Mya (Stümpel et al., 2016). However, these authors propose that the valley of Göksu River south-west of the Anatolian Diagonal formed the natural barrier for the vipers adapted to the life in high altitudes, which was probably not true for the eurytopic rat snakes. Nevertheless, similar patterns of the east-west Anatolian split have been described in numerous other reptilian taxa (see Fritz et al., 2008; Kyriazi et al., 2008; Kornilios et al., 2012; Ahmadzadeh et al., 2013; Jandzik, Avcı & Gvoždík, 2013; Jandzik et al., 2018; Kapli et al., 2013; Skourtanioti et al., 2016).

*Elaphe sauromates* s. s. then presumably survived the Pleistocene climatic oscillations in refugia located mainly in Anatolia, Crimea, and/or the southern Balkans, where the highest haplotype diversity was recorded (see Figs. 1B and 1C). Subsequently in the Late Pleistocene, *E. sauromates* s. s. reoccupied the territories of eastern Europe concomitant with dispersing north of the steppe and semi-desert habitats and the Black Sea regression (Ratnikov, 2014), similarly as proposed for the snake *Dolichophis caspius* (Nagy et al., 2010) and lizards *Lacerta viridis* (Marzahn et al., 2016), *Pseudopus apodus* (Jandzik et al., 2018), and *Podarcis tauricus* (Psonis et al., 2018). Before the terminal phase of the last Pleistocene glaciation, *E. sauromates* already lived in the Crimean Mountains as confirmed by the fossil record (Ratnikov, 2015), although remnants of *E. quatuorlineata–sauromates* from the Pleistocene and Holocene are not known from the East European Plain (Ratnikov, 2002). Interestingly, Mazanaeva & Tuniyev (2011) proposed that *E. sauromates* and other Mediterranean faunal elements might have colonized the Great Caucasus from the North through the Kuma–Manych Strait during the last phases of the Pleistocene. Our lack of samples from the south-eastern parts of *E. sauromates* s. s. range does not allow us to confirm or reject this scenario. Colonization of the Transcaspia and Central Asia is probably very recent as the region is inhabited by the common northern *E. sauromates* s. s. haplotypes (Figs. 1A–1C) and might have coincided with the Late Pleistocene or even the Early Holocene regression of the Caspian Sea (see Krijgsman et al., 2018).

The significantly lower genetic diversity of *E. urartica* sp. nov. (only two haplotypes in *COI*, three in *ND4*, one to three in the nDNA genes; Figs. 1 and 3) does not provide enough information for drawing viable biogeographic scenarios. The species probably survived the Pleistocene glaciations in one, or a few, Transcaucasian or east-Anatolian refugia (Kahle et al., 2011; Kornilios et al., 2011; Jandzik et al., 2018) and spread relatively fast to the north-eastern Caucasus and southern Anatolia. A very similar situation with low genetic variation in the same region was observed in an anguid lizard *Pseudopus apodus* (Jandzik et al., 2018). Additional samples from Iran, central Anatolia, and the Levant will be necessary to learn more about the history of this species. In particular, one might expect surprises from the Levantine region given that it hosts many endemic taxa and lineages, and several Anatolian species have their subspecies or sister species located in the region, for example, *Natrix tessellata* complex (Guicking, Joger & Wink, 2009), the *L. trilineata* complex (Ahmadzadeh et al., 2013), *Z. hohenackeri* (Jandzik, Avcı & Gvoždík, 2013), the *Ablepharus kitaibelii* complex (Skourtanioti et al., 2016), *Xerotyphlops vermicularis* (Kornilios, 2017), and the *Mediodactylus kotschyi* complex (Kotsakiozi et al., 2018).

## Conclusions

Here we studied the biogeography of the rat snake *E. sauromates* from the Balkans, Anatolia, Caucasus, and Ponto-Caspian region using both molecular and morphological data. We found that the taxon is, in fact, comprised of two distinct evolutionary lineages and the cryptic lineage represents a new species that we name *E. urartica* sp. nov. Both species split from their common ancestor around the Miocene-Pliocene boundary and their recent genetic structure was mainly influenced by Pleistocene climatic oscillations.

## Supplemental Information

10.7717/peerj.6944/supp-1 Supplemental Information 1 Supplementary Information.

10.7717/peerj.6944/supp-2 Supplemental Information 2 Phylogenetic relationships of *Elaphe quatuorlineata*, *E. sauromates*, and *E. urartica* sp. nov. reconstructed using Bayesian inference of concatenated *COI* and *ND4* sequences. The numbers above the branches represent Bayesian Posterior probabilities showing the branch support.

10.7717/peerj.6944/supp-3 Supplemental Information 3 Phylogenetic relationships of *Elaphe quatuorlineata*, *E. sauromates*, and *E. urartica* sp. nov. reconstructed using Maximum Likelihood tree analysis of concatenated *COI* and *ND4* sequences. The numbers above the branches represent bootstraps showing the branch support.

10.7717/peerj.6944/supp-4 Supplemental Information 4 Alignments of mitochondrial and nuclear sequences used in this study.

## Additional Information and Declarations

### Field Study Permissions

The following information was supplied relating to field study approvals (i.e., approving body and any reference numbers): Field study permits were issued by the Republic of Turkey Ministry of Forest and Water Affairs (No. B.23.0.DMP.0.15.01–510–38417), by the Crimean authority under the title “Study of Biodiversity and Landscape Structure of the Southeastern Crimea, monitoring of Biotic and Abiotic Components of the Regional Ecosystems” (FASO: AAA–A16–116022510087–5) and by “Study of the structure and dynamics of land ecosystems in various climatic zones” No. AAAA–A19–119012490044–3).

### DNA Deposition

The following information was supplied regarding the deposition of DNA sequences: The group of mitochondrial and nuclear DNA sequences described here is accessible via GenBank accession numbers MK640236 to MK640422.

### Data Availability

The following information was supplied regarding data availability: All sequences are available in the GenBank database. Accession numbers for the DNA sequences included in this study are provided in Table 1. The sequences are also available as a Supplemental File.

### New Species Registration

The following information was supplied regarding the registration of a newly described species: Publication LSID: urn:lsid:zoobank.org:pub:F0BF1D63-7BD5-4340-85A0-E044CC56CD5B. *Elaphe urartica* Jablonski, Kukushkin, Avcı, Bunyatova, Ilgaz, Tuniyev et Jandzik sp. nov. LSID: urn:lsid:zoobank.org:act:A8F964EB-FD8E-4EAB-9549-DECD0648075C.
