# Supplementary material for: The biogeography of Elaphe sauromates (Pallas, 1814), with a description of a new rat snake species
Source: PeerJ. 2019 May 28;7:e6944. doi: 10.7717/peerj.6944 (PMC6544014; doi:10.7717/peerj.6944)

**Supplementary Information**

**Comments on the nomenclatural acts**

In the description of *Elaphe urartica* sp. nov. we declare three historical names *nomina dubia*: *Coluber cereus* Dwigubsky, 1832, *C. fulvus* Dwigubsky, 1832, and *C. taeniothys* Fischer von Waldheim, 1832. These names have been cited in literature as synonyms of *E. sauromates* possibly originating from the recent distribution range of *E. urartica* sp. nov. (e.g. Nikolsky 1916, Helfengberger 2001; note that both cite the Fisher von Waldheim’s name erroneously as *C. thaeniothys*), although none of the original descriptions indicated the locality of origin of the respective material. Regrettably, all material, which the names and descriptions refer to, has been lost in the first half of the 20^th^ century (i.e. to our best knowledge it is not present in any of the main zoological museums of the former USSR).

Hohenacker (1831) described his collection of several *Coluber* sp. specimens from the Caucasus (“provinces méridionales du Caucasus” = Transcaucasia) without proper specific identification (he only used numbers to list them) or more detailed locality description. Based on part of Hohenacker’s 1831 material (Nos. 15 and 18) and translating his description from French into Russian, Dwigubsky (1832) named two species, *C. cereus* (corresponding to Hohenacker’s No. 15) and *C. fulvus* (corresponding to Hohenacker’s No. 18). Interestingly, Dwigubsky admitted in a footnote that he only did so based on Hohenacker’s description, without having the opportunity to study the material or even see the images (Dwigubsky 1832, p. 25), thus he could not be certain of the snake’s proper identification. He also did not name the precise locality, only generally mentions “different places in the Caucasus”. Additionally, some of the characteristics of both named taxa lie clearly outside the normal variation of *E. sauromates* (and of *E. urartica* sp. nov.), e.g. 240 ventrals in *C. cereus* and dark-red belly in *C. fulvus*.

Only a few months after Dwigubsky’s (1832) publication, Fischer von Waldheim (1832) published a description of *C. taeniothys* Fischer von Waldheim, 1832, corresponding to Hohenacker’s Coluber No. 15. (and thus to Dwigubsky’s *C. cereus* as well), referring to the locality as “environs du Caucase”, but without selecting any type material or listing more details on the locality. In contrast to Dwigubsky (1832), Fischer von Waldheim (1832) described *C. taeniothys* as having 204 ventrals (meaning either Dwigubsky’s or Fischer von Waldheim’s description is erroneous). The only case known to us when this name has since been used was by Hohenacker (1837), who listed it among material collected in the Transcaucasian provinces of Karabach, Schirwan and Talysch. The locality he provides for this species is Helenendorf (renamed to Khanlar in 1938 and to Göygöl in 2008, Azerbaijan).

Due to the confusing history of all three names, inconsistencies in the material descriptions, lack of details on type localities, missing type material, and the uncertainty of proper identification due to the aforementioned disparities, we declare all three above mentioned names *nomina dubia* (ICZN 2012).

**References**

Dwigubsky I. (1832). Opyt estestvennoyi istorii vsekh zhivotnykh Rossijskoyi Imperii. Tom 4. Gady, ili zhivotnye presmykayuschiesya [Essay the Natural History of Animals from the Russian Empire. V. 4. Herptiles, or Crawling animals]. Moscow: Moscow University Printing House. 48 p. + 12 tables with illustrations

Fischer G. (1832). Notices sur les nouvelles acquisitions de la Société de l’année 1832 // Bulletin de la Société Impériale des Naturalistes de Moscou. Tome 4. Moscou: De l’Imprimérie de l’Université Impériale. P. 572–595.

Helfenberger N. (2001). Phylogenetic relationships of Old World ratsnakes based on visceral organ topography, osteology, and allozyme variation // Russian Journal of Herpetology. 2001. V. 8 (Supplement). 64 p.

Hohenacker F. (1831). Notices sur quelques objets d`histoire naturelle des provinces méridionalis du Caucase que l`on peut procurer par les roins de Mr. Hohenacker // Bulletin de la Société Impériale des Naturalistes de Moscou. Tome 3. Moscou: De l’Imprimérie de l’Université Impériale. P. 363–378.

Hohenacker R. F. (1837). Enumeratio animalium quae in provinciis transcaucasicis Karabach, Shirwan et Talysch nec non in territorio Elisabethopolensi observavit // Bulletin de la Société Impériale des Naturalistes de Moscou. Tome 7, No. 6. Moscou: Imprimeur de l’Académie Impériale médico-chirurgicale. P. 136–147.

ICZN (2012). International Code of Zoological Nomenclature, online. The International Trust for Zoological Nomenclature, London. Available on: <http://www.iczn.org/iczn/index.jsp>

Nikolsky A. M. (1916). Presmykayuschiesya (Reptilia). Tom II. Ophidia / Fauna Rossii I sopredel’nykh stran [Reptiles (Reptilia). V. II. Ophidia / Fauna of Russia and adjacent countries] / N. V. Nasonov (ed.). Petrograd: Publishing House of Imperial Academy of Sciences. 350 p. + 8 tables with illustrations

Table S1. List of primers used in the amplification and sequencing of the amplified gene fragments, with the corresponding source and PCR conditions. Primers orientation (OR); F=forwards, R=reverse.

| **Locus** | **Primer name** | **OR** | **Primer sequence (5’- 3’)** | **Primer source** | **PCR conditions** |
| --- | --- | --- | --- | --- | --- |
| *COI* | COI(+)deg1 | F | AAGCTTCTGACTNCTACCACCNGC | Utiger et al. (2002) | 94º(3’);94º(60”), 57º(60”), 72º (60”) x 35;72º(10’) |
|  | COI(-)bdeg | R | ATTATTGTTGCYGCTGTRAARTAGGCTCG | Utiger et al. (2002) |  |
| *ND4* | ND4 | F | CACCTATGACTACCAAAAGCTCATGTAGAAGC | Arévalo et al. (1994) | 94º(3’);94º(60”), 54º(30”), 72º (60”) x 35;72º(5’) |
|  | Leu | R | CATTACTTTTACTTGGAATTTGCACCA | Arévalo et al. (1994) |  |
| *C-MOS* | S77 | F | CATGGACTGGGATCAGTTATG | Lawson et al. (2005) | 94º(7’);94º(40”), 48º(30”), 72º (60”) x 40;72º(7’) |
|  | S78 | R | CCTTGGGTGTGATTTTCTCACCT | Lawson et al. (2005) |  |
| *MC1R* | MC1R-F | F | GGCNGCCATYGTCAAGAACCGGAACC | Pinho et al. (2009) | 94º(5’);94º(30”), 52º(45”), 72º (90”) x 40;72º(5’) |
|  | MC1R-R | R | CTCCGRAAGGCRTAAATGATGGGGTCCAC | Pinho et al. (2009) |  |
| *RAG1* | R13 | F | TCTGAATGGAAATTCAAGCTGTT | Groth and Barrowclough (1999) | 94º(5’);94º(40”), 63º(30”), 72º (60”) x 39;72º(10’) |
|  | R18 | R | GATGCTGCCTCGGTCGGCCACCTTT | Groth and Barrowclough (1999) |  |
| *PRLR* | PRLR_f1 | F | GACARYGARGACCAGCAACTRATGCC | Townsend et al. (2008) | 94º(5’);94º(40”), 50º(30”), 72º (60”) x 40;72º(7’) |
|  | PRLR_r3 | R | GACYTTGTGRACTTCYACRTAATCCAT | Townsend et al. (2008) |  |

**References**

Arévalo, E., Davis, S. K. & Sites, J. W. (1994). Mitochondrial DNA Sequence Divergence and Phylogenetic Relationships among Eight Chromosome Races of the *Sceloporus grammicus* Complex (Phrynosomatidae) in Central Mexico. Systematic Biology 43: 387-418.

Pinho C, Rocha S, Carvalho BM, Lopes S, Mourao S et al. (2009). New primers for the amplification and sequencing of nuclear loci in a taxonomically wide set of reptiles and amphibians. Conserv. Genet Resour. 2: 181-185.

Groth JG, Barrowclough GF (1999) Basal Divergences in Birds and the Phylogenetic Utility of the Nuclear RAG-1 Gene. Mol Phylogenet Evol 12: 115-123.

Lawson, R., Slowinski, J. B., Crother, B. I. & Burbrink, F. T. (2005). Phylogeny of the Colubroidea (Serpentes): new evidence from mitochondrial and nuclear genes. Mol Phylogenet Evol 37: 581-601.

Townsend, T. M., Alegre, R. E., Kelley, S. T., Wiens, J.J. & Reeder, T. W. (2008). Rapid development of multiple nuclear loci for phylogenetic analysis using genomic resources: An example from squamate reptiles. Mol Phylogenet Evol 47: 129-142.

Utiger U., Helfenberger N., Schätti B., Schmidt C., Ruf M. & Ziswiler V. (2002). Molecular systematics and phylogeny of Old World and New World ratsnakes, *Elaphe* auct., and related genera (Reptilia, Squamata, Colubridae). Russian Journal of Herpetology 9 (2): 105–124.

Table S2. Explanation of measures used in morphological analyses.

| Measure/Count | Description |
| --- | --- |
| Snout-vent length | The longitudinal length from the snout tip to the posterior margin of the posterior-most ventral scale |
| Total length | Sum of Snout-vent length and tail length |
| Head length | Longitudinal distance between the tip of the snout and posterior edge of the posterior labial |
| Head width (inter-ocular) | Transverse distance between the head edges in the level of eye axis |
| Pileus length | Longitudinal distance between the tip of the snout and posterior edge of the occipital plate |
| Pileus width | Transverse distance between the lateral edges of the parietal plates |
| Rostrum height | Height of the rostrum |
| Rostrum width | Transverse width of the rostrum |
| Inter-nostril width | Transverse distance between the nostrils |
| Eye diameter | Longitudinal diameter of the left eye |
| Plate lengths/widths | Maximum longitudinal/transverse dimension of the plate on the left side of the head |

Table S3. Frequencies of selected scale counts in *E. sauromates* and *E. urartica* sp. nov.

| Character | ***Elaphe sauromates*** | | | | | | ***Elaphe urartica* sp. nov.** | | | | | |
| --- | --- | --- | --- | --- | --- | --- | --- | --- | --- | --- | --- | --- |
|  | Males | | | Females | | | Males | | | Females | | |
|  | N | Range | Frequency | N | Range | Frequency | N | Range | Frequency | N | Range | Frequency |
| Preventrals | 22 | 0-3 |  | 11 | 1-2 |  | 12 | 1-2 |  | 10 | 0-3 |  |
| - Freq. of 0 |  |  | 13.8 % (4) |  |  | 0 |  |  | 0 |  |  | 10.0 % (1) |
| - Freq. of 1 |  |  | 31.0 % (9) |  |  | 63.6 % (7) |  |  | 83.3 % (10) |  |  | 60.0 % (6) |
| - Freq. of 2 |  |  | 20.7 % (6) |  |  | 36.4 % (4) |  |  | 16.7 % (2) |  |  | 20.0 % (2) |
| - Freq. of 3 |  |  | 10.3 % (3) |  |  | 0 |  |  | 0 |  |  | 10.0 % (1) |
| Rows of dorsals one head length posterior to the head | 17 | 21-25 |  | 6 | 21-27 |  | 12 | 23-25 |  | 10 | 25 |  |
| - Freq. of 21 |  |  | 5.9 % (1) |  |  | 16.7 % (1) |  |  | 0 |  |  | 0 |
| - Freq. of 22 |  |  | 5.9 % (1) |  |  | 0 |  |  | 0 |  |  | 0 |
| - Freq. of 23 |  |  | 23.5 % (4) |  |  | 0 |  |  | 8.3 % (1) |  |  | 0 |
| - Freq. of 24 |  |  | 17.63 % (3) |  |  | 16.7 % (1) |  |  | 16.7 % (2) |  |  | 0 |
| - Freq. of 25 |  |  | 47.1 % (8) |  |  | 50.0 % (3) |  |  | 75.0 % (9) |  |  | 100 % (10) |
| - Freq. of 26 |  |  | 0 |  |  | 0 |  |  | 0 |  |  | 0 |
| - Freq. of 27 |  |  | 0 |  |  | 16.7 % (1) |  |  | 0 |  |  | 0 |
| Rows of dorsals at midbody | 26 | 23-25 |  | 16 | 24-25 |  | 19 | 23-25 |  | 13 | 24-25 |  |
| - Freq. of 23 |  |  | 23.1 % (6) |  |  | 0 |  |  | 21.1 % (4) |  |  | 0 |
| - Freq. of 24 |  |  | 3.8 % (1) |  |  | 12.5 % (2) |  |  | 5.3 % (1) |  |  | 7.7 % (1) |
| - Freq. of 25 |  |  | 73.1 % (23) |  |  | 87.5 % (14) |  |  | 73.7 % (14) |  |  | 92.3 % (12) |
| Rows of dorsals one head length posterior to the cloaca | 17 | 18-19 |  | 6 | 19-21 |  | 12 | 19 |  | 10 | 18-19 |  |
| - Freq. of 18 |  |  | 5.9 % (1) |  |  | 0 |  |  | 0 |  |  | 10.0 % (1) |
| - Freq. of 19 |  |  | 94.1 % (16) |  |  | 50.0 % (3) |  |  | 100 % (12) |  |  | 90.0 % (9) |
| - Freq. of 20 |  |  | 0 |  |  | 33.3 % (2) |  |  | 0 |  |  | 0 |
| - Freq. of 21 |  |  | 0 |  |  | 16.7 % (1) |  |  | 0 |  |  | 0 |
| Preoculars | 23 | 1-3 |  | 13 | 1-2 |  | 19 | 1-3 |  | 13 | 1-2 |  |
| - Freq. of 1 |  |  | 21.7 % (5) |  |  | 15.4 % (2) |  |  | 42.1 % (8) |  |  | 69.2 % (9) |
| - Freq. of 2 |  |  | 65.2 % (15) |  |  | 84.6 % (11) |  |  | 52.6 % (10) |  |  | 30.8 % (4) |
| - Freq. of 3 |  |  | 13.0 % (3) |  |  | 0 |  |  | 5.3 % (1) |  |  | 0 |
| Loreals (tip of) | 19 | 1-3 |  | 9 | 1-3 |  | 12 | 1-2 |  | 10 | 1-2 |  |
| - Freq. of 1 |  |  | 31.6 % (6) |  |  | 11.1 % (1) |  |  | 91.7 % (11) |  |  | 90.0 (9) |
| - Freq. of 2 |  |  | 52.6 % (10) |  |  | 66.7 % (6) |  |  | 8.3 % (2) |  |  | 10.0 % (1) |
| - Freq. of 3 |  |  | 15.8 % (3) |  |  | 22.2 % (2) |  |  | 0 |  |  | 0 |
| Postoculars | 21 | 1-2 |  | 11 | 2 |  | 19 | 1-2 |  | 13 | 2-3 |  |
| - Freq. of 1 |  |  | 4.8 % (1) |  |  | 0 |  |  | 5.3 % (1) |  |  | 0 |
| - Freq. of 2 |  |  | 95.2 % (20) |  |  | 100 % (11) |  |  | 94.7 % (18) |  |  | 84.6 % (11) |
| - Freq. of 3 |  |  | 0 |  |  | 0 |  |  | 0 |  |  | 15.4 % (2) |
| Temporals | 23 | 1-3 |  | 15 | 2-3 |  | 19 | 2 |  | 13 | 2-3 |  |
| - Freq. of 1 |  |  | 4.3 % (1) |  |  | 0 |  |  | 0 |  |  | 0 |
| - Freq. of 2 |  |  | 65.2 % (15) |  |  | 60.0 % (9) |  |  | 100 % (19) |  |  | 76.9 % (10) |
| - Freq. of 3 |  |  | 20.4 % (7) |  |  | 40.0 % (6) |  |  | 0 |  |  | 23.1 % (3) |
| Posttemporals | 23 | 2-5 |  | 15 | 2-5 |  | 19 | 2-4 |  | 13 | 3-4 |  |
| - Freq. of 2 |  |  | 17.4 % (4) |  |  | 26.7 % (4) |  |  | 10.5 % (2) |  |  | 0 |
| - Freq. of 3 |  |  | 21.7 % (5) |  |  | 33.3 % (5) |  |  | 31.6 % (6) |  |  | 38.5 % (5) |
| - Freq. of 4 |  |  | 56.5 % (13) |  |  | 26.7 % (4) |  |  | 57.9 % (11) |  |  | 61.5 % (8) |
| - Freq. of 5 |  |  | 4.3 % (1) |  |  | 13.3 % (2) |  |  | 0 |  |  | 0 |
| Labials | 26 | 7-9 |  | 16 | 8-10 |  | 18 | 8 |  | 13 | 8-9 |  |
| - Freq. of 7 |  |  | 3.8 % (1) |  |  | 0 |  |  | 0 |  |  | 0 |
| - Freq. of 8 |  |  | 92.3 % (24) |  |  | 75 % (12) |  |  | 100 % (18) |  |  | 92.3 % (12) |
| - Freq. of 9 |  |  | 3.8 % (1) |  |  | 18.8 % (3) |  |  | 0 |  |  | 7.7 % (1) |
| - Freq. of 10 |  |  | 0 |  |  | 6.3 % (1) |  |  | 0 |  |  | 0 |
| Labials touching the eye | 23 | 4^th^-6^th^ |  | 13 | 4^th^-7^th^ |  | 7 | 4^th^-5^th^ |  | 7 | 4^th^-6^th^ |  |
| - 4^th^+5^th^ |  |  | 95.7 % (22) |  |  | 84.6 % (11) |  |  | 100 % (7) |  |  | 85.7 % (6) |
| - 5^th^+6^th^ |  |  | 4.3 % (1) |  |  | 7.7 % (1) |  |  | 0 |  |  | 14.3 % (1) |
| - 6^th^+7^th^ |  |  | 0 |  |  | 7.7 % (1) |  |  | 0 |  |  | 0 |
| Sublabials | 26 | 9-12 |  | 15 | 9-12 |  | 15 | 10-13 |  | 11 | 10-12 |  |
| - Freq. of 9 |  |  | 7.7 % (2) |  |  | 6.7 % (1) |  |  | 0 |  |  | 0 |
| - Freq. of 10 |  |  | 30.8 % (8) |  |  | 40.0 % (6) |  |  | 13.3 % (2) |  |  | 36.4 % (4) |
| - Freq. of 11 |  |  | 42.3 % (11) |  |  | 46.7 % (7) |  |  | 73.3 % (11) |  |  | 54.5 % (6) |
| - Freq. of 12 |  |  | 19.2 % (5) |  |  | 6.7 % (1) |  |  | 6.7 % (1) |  |  | 9.1 % (1) |
| - Freq. of 13 |  |  | 0 |  |  | 0 |  |  | 6.7 % (1) |  |  | 0 |
| Gulars between posterior labials | 15 | 10-16 |  | 6 | 13-15 |  | 12 | 13-16 |  | 10 | 11-16 |  |
| - Freq. of 10 |  |  | 6.7 % (1) |  |  | 0 |  |  | 0 |  |  | 0 |
| - Freq. of 11 |  |  | 0 |  |  | 0 |  |  | 0 |  |  | 10.0 % (1) |
| - Freq. of 12 |  |  | 6.7 % (1) |  |  | 0 |  |  | 0 |  |  | 10.0 % (1) |
| - Freq. of 13 |  |  | 20.0 % (3) |  |  | 50.0 % (3) |  |  | 50.0 % (6) |  |  | 30.0 % (3) |
| - Freq. of 14 |  |  | 26.7 % (4) |  |  | 16.7 % (1) |  |  | 0 |  |  | 30.0 % (3) |
| - Freq. of 15 |  |  | 33.3 % (5) |  |  | 33.3 % (2) |  |  | 33.3 % (4) |  |  | 20.0 % (2) |
| - Freq. of 16 |  |  | 0 |  |  | 0 |  |  | 16.7 % (2) |  |  |  |
| Gulars between anterior intermaxillars | 20 | 0-2 |  | 10 | 1 |  | 12 | 1-2 |  | 10 | 1-2 |  |
| - Freq. of 0 |  |  | 5.0 % (1) |  |  | 0 |  |  | 0 |  |  | 0 |
| - Freq. of 1 |  |  | 80.0 % (16) |  |  | 100 % (10) |  |  | 50.0 % (6) |  |  | 30.0 % (3) |
| - Freq. of 2 |  |  | 15.0 % (3) |  |  | 0 |  |  | 50.0 % (6) |  |  | 70.0 % (7) |
| Gulars between posterior intermaxillars | 20 | 0-4 |  | 10 | 1-4 |  | 12 | 2-4 |  | 10 | 2-5 |  |
| - Freq. of 0 |  |  | 5.0 % (1) |  |  | 0 |  |  | 0 |  |  | 0 |
| - Freq. of 1 |  |  | 0 |  |  | 10.0 % (1) |  |  | 0 |  |  | 0 |
| - Freq. of 2 |  |  | 50.0 % (10) |  |  | 40.0 % (4) |  |  | 58.3 % (7) |  |  | 80.0 % (8) |
| - Freq. of 3 |  |  | 35.0 % (7) |  |  | 40.0 % (4) |  |  | 33.3 % (4) |  |  | 10.0 % (1) |
| - Freq. of 4 |  |  | 10.0 % (2) |  |  | 10.0 % (1) |  |  | 8.3 % (1) |  |  | 0 |
| - Freq. of 5 |  |  | 0 |  |  | 0 |  |  | 0 |  |  | 10.0 % (1) |

Table S4. Measures of juvenile (after the first hibernation) *Elaphe urartica* sp. nov. from Armenia.

|  | **Males** | | **Females** | |
| --- | --- | --- | --- | --- |
|  | N = 3 | | N = 5 | |
| Snout-vent length (SVL) | 402–440 | 421+16 | 412–451 | 427+10 |
| Tail length (TL) | 85–101 | 94+5 | 73–84 | 77+2 |
| Total length | 487–541 | 515+11 | 473–535 | 504+12 |
| SVL/TL | 4.36–4.73 | 4.49+0.12 | 5.37–5.76 | 5.55+0.07 |
| Head length (HL) | 17.5–18.0 | 17.8+0.2 | 16.85–18.4 | 17.4+0.3 |
| SVL/HL | 22.97–24.72 | 23.7+0.53 | 23.67–25.51 | 24.5+0.34 |
| Head width (inter-ocular) | 7.8–8.2 | 8.0+0.1 | 7.7–8.0 | 7.8+0.1 |
| Pileus length | 16.2–16.6 | 16.4+0.1 | 15.1–16.2 | 15.7+0.2 |
| Pileus width | 8.7–9.8 | 9.1+0.3 | 8.7–9.3 | 8.9+0.1 |
| Rostrum height | 3.5–3.5 | 3.5+0.1 | 2.8–3.6 | 3.3+0.1 |
| Rostrum width | 4.3–4.5 | 4.4+0.1 | 4.0–4.6 | 4.3+0.1 |
| Inter-nostril width | 4.4–4.9 | 4.7+0.1 | 4.3–4.6 | 4.4+0.1 |
| Eye diameter | 3.4–3.6 | 3.5+0.1 | 3.4–3.7 | 3.5+0.1 |
| Supraocular plate width | 2.9–3.2 | 3.0+0.1 | 2.8–3.0 | 2.9+0.1 |
| Frontal plate length | 4.8–5.4 | 5.1+0.2 | 5.1–5.5 | 5.2+0.1 |
| Frontal plate width | 3.9–4.2 | 4.0+0.1 | 3.5–4.1 | 3.8+0.1 |
| Anterior intermaxillary length | 4.5–5.1 | 4.8+0.2 | 4.5–4.9 | 4.7+0.1 |
| Posterior intermaxillary length | 4.1–4.5 | 4.3+0.1 | 2.9–4.1 | 3.5+0.2 |

Fig. S1. Phylogenetic relationships of *Elaphe quatuorlineata*, *E. sauromates*, and *E. urartica* sp. nov. reconstructed using Bayesian inference of concatenated *COI* and *ND4* sequences. The numbers above the branches represent Bayesian Posterior probabilities showing the branch support.


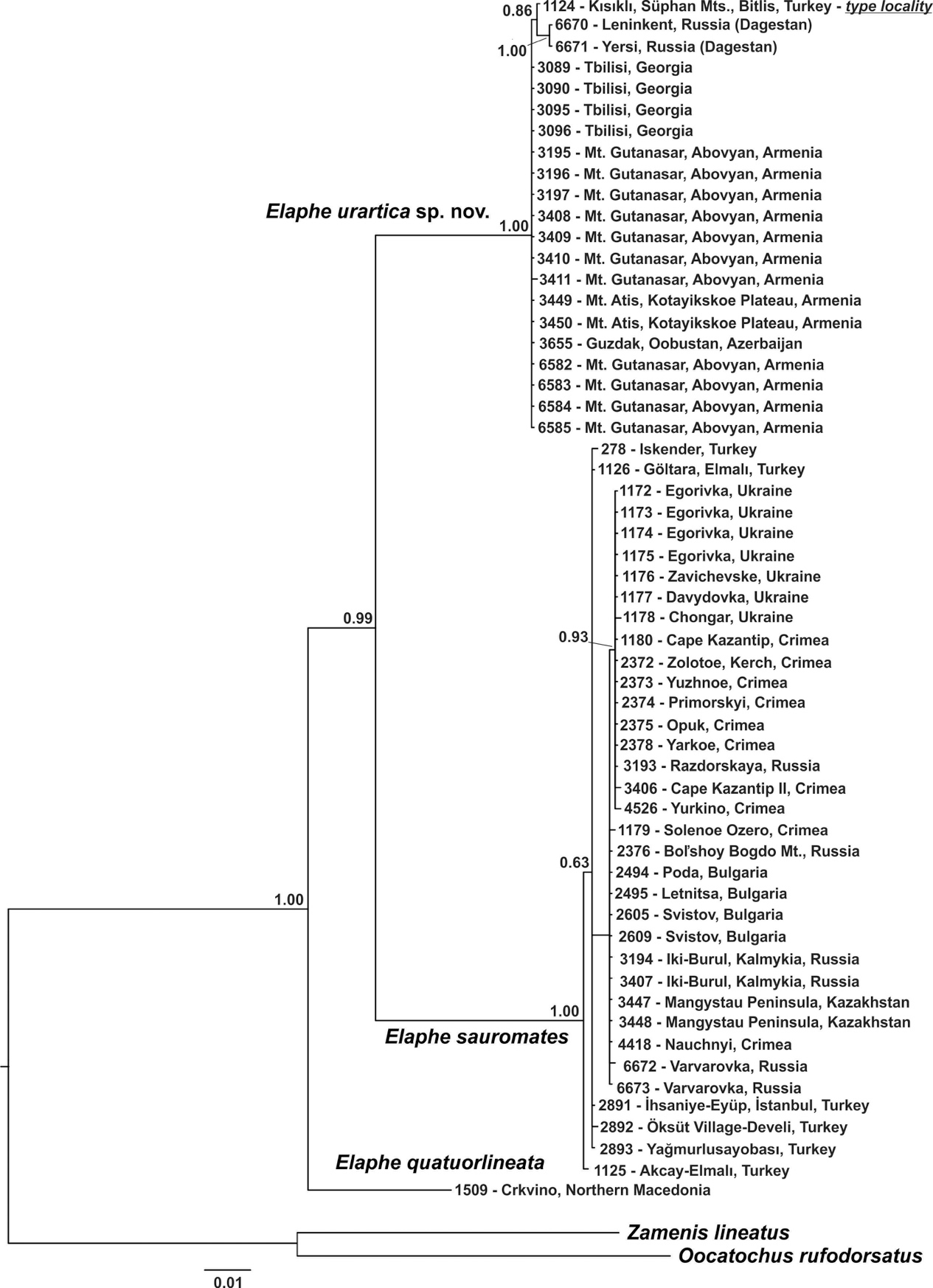


Fig. S2. Phylogenetic relationships of *Elaphe quatuorlineata*, *E. sauromates*, and *E. urartica* sp. nov. reconstructed using Maximum Likelihood tree analysis of concatenated *COI* and *ND4* sequences. The numbers above the branches represent bootstraps showing the branch support.


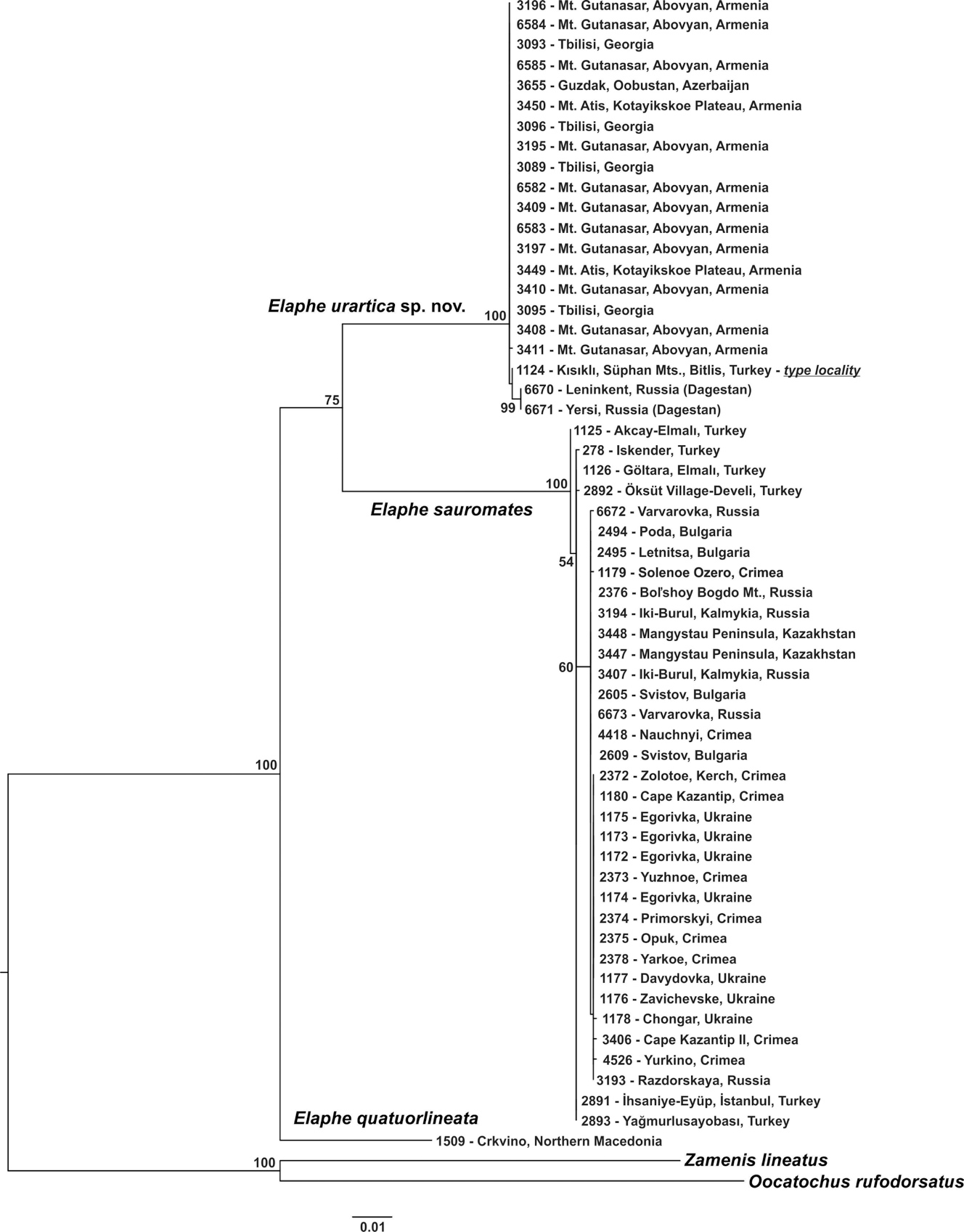

Supplement: Supplemental Information 1 [file peerj-07-6944-s001.docx]
