# Supplementary figures and images for: The biogeography of Elaphe sauromates (Pallas, 1814), with a description of a new rat snake species
Source: PeerJ. 2019 May 28;7:e6944. doi: 10.7717/peerj.6944 (PMC6544014; doi:10.7717/peerj.6944)

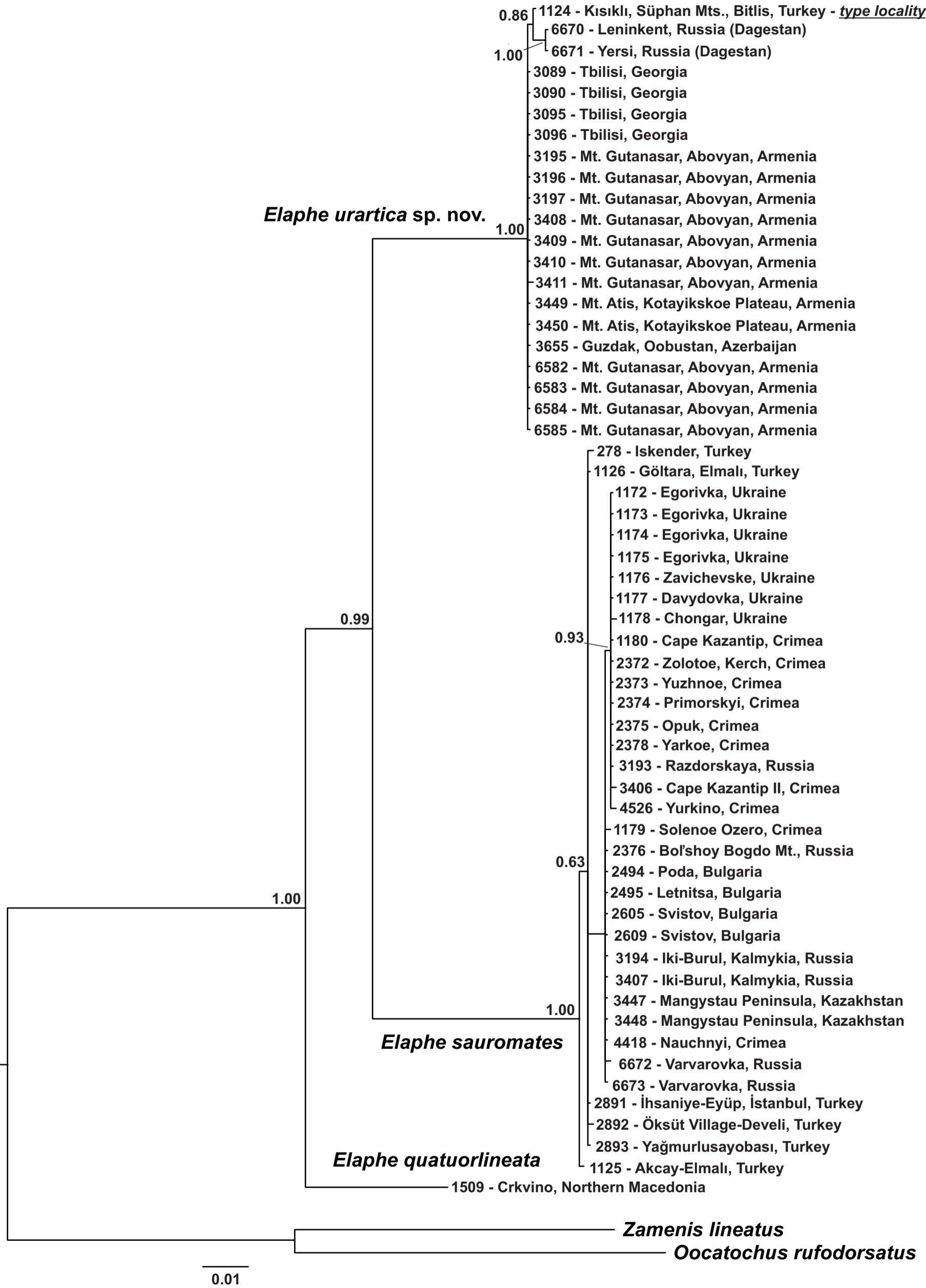

Supplement: Supplemental Information 2 — The numbers above the branches represent Bayesian Posterior probabilities showing the branch support. [file peerj-07-6944-s002.pdf]

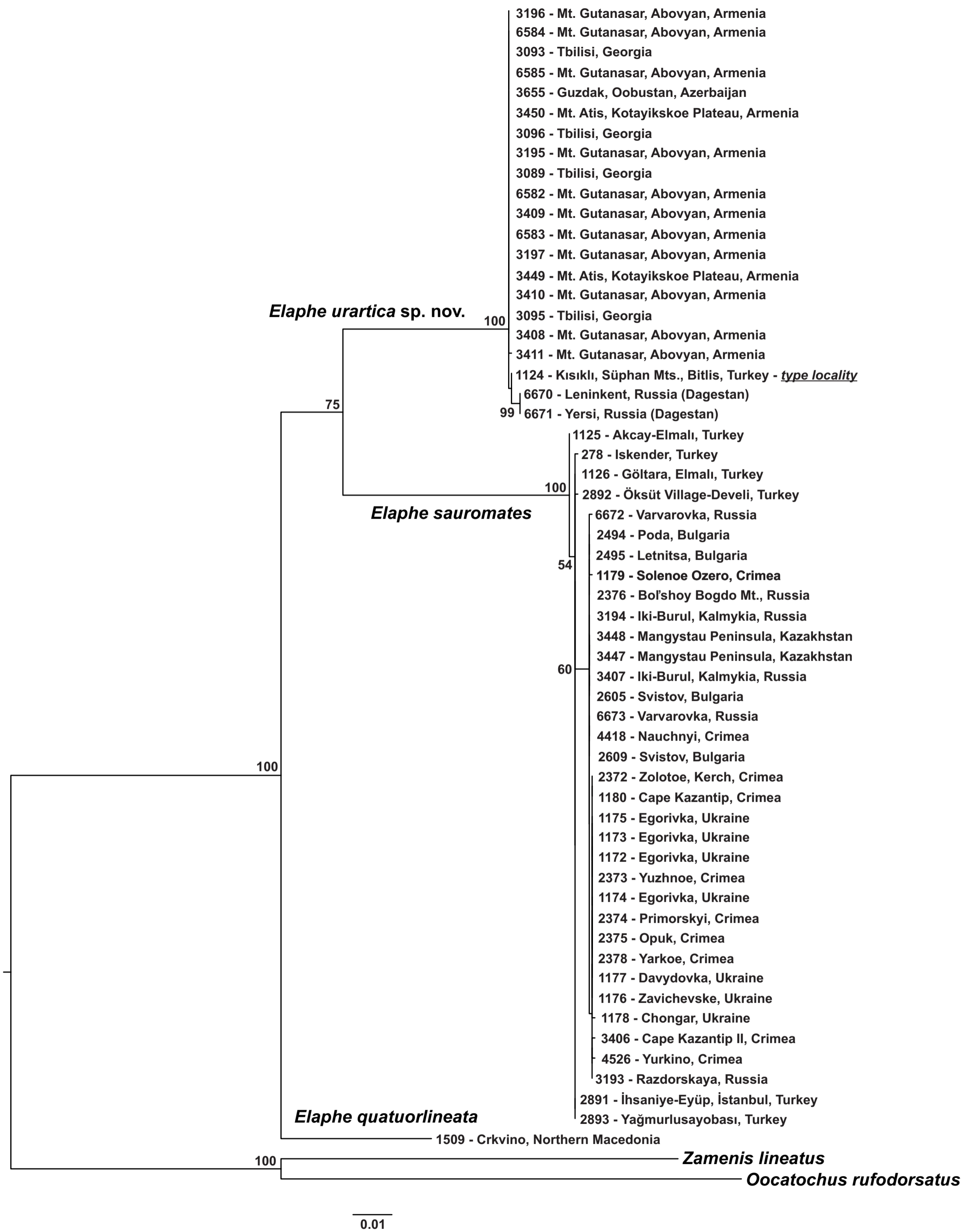

Supplement: Supplemental Information 3 — The numbers above the branches represent bootstraps showing the branch support. [file peerj-07-6944-s003.pdf]
